# Clinical outcomes and treatment patterns of older adults with dementia-related psychosis by dementia type in the United States

**DOI:** 10.1186/s12877-022-03489-3

**Published:** 2022-10-06

**Authors:** Joan Forns, Heather E. Danysh, Lisa J. McQuay, Mary Ellen Turner, Colleen Dempsey, Mary S. Anthony, George Demos, J. Bradley Layton

**Affiliations:** 1 Pharmacoepidemiology and Risk Management, RTI Health Solutions, Barcelona, Spain; 2 Pharmacoepidemiology and Risk Management, RTI Health Solutions, Waltham, MA USA; 3 Pharmacoepidemiology and Risk Management, RTI Health Solutions, Research Triangle Park, NC USA; 4 Drug Safety and Pharmacovigilance, ACADIA Pharmaceuticals Inc, Princeton, NJ USA; 5 Drug Safety and Pharmacovigilance, ACADIA Pharmaceuticals Inc, San Diego, CA USA

**Keywords:** Alzheimer’s disease, Antipsychotics, Dementia with Lewy bodies, Frontotemporal dementia, Infections, Healthcare utilization, Medicare, Mortality, Parkinson’s disease dementia, Vascular dementia

## Abstract

**Background:**

Little is known about the incidence of clinical events and treatment patterns among older adults with dementia-related psychosis. Given that dementia-related psychosis comprises various dementia types, this study describes the incidence of clinical events and treatment patterns by dementia type after patients with dementia are diagnosed with psychosis.

**Methods:**

Adults aged ≥ 65 years with dementia and newly diagnosed with psychosis were identified in US Medicare claims during 2013–2018. Baseline characteristics were evaluated at the time of the psychosis diagnosis. After the initial psychosis diagnosis, incidence rates (IRs) of clinical events (e.g., falls/fractures, infections, healthcare utilization), mortality, and patterns of antipsychotic treatment were described for each dementia type (Alzheimer’s disease [AD], Parkinson’s disease dementia [PDD], dementia with Lewy bodies [DLB], frontotemporal dementia [FTD], vascular dementia [VD], and unspecified dementia). Daily mean cumulative counts were estimated to describe the incidence of recurrent events over time. Mortality was described using Kaplan–Meier survival curves.

**Results:**

We identified 484,520 patients with dementia-related psychosis: mean age, 84 years (standard deviation, 7.8); female, 66%. At the time of psychosis diagnosis, the most prevalent type of dementia was unspecified dementia (56%), followed by AD (31%), VD (12%), PDD (10%), DLB (3%), and FTD (< 1%), and most patients had scores indicating severe illness on the Charlson Comorbidity Index (71%) and frailty index (62%). Across all dementia types, IRs (per 100 person-years) were high for emergency department visits, oral anti-infective use, and urinary tract infections after the initial psychosis diagnosis. Patients with DLB had the highest incidence of most clinical outcomes. After 1 year of follow-up, the cumulative probability of death was about 30% for all dementia types, and after 5 years, was about 80% among patients with DLB, VD, AD, or PDD and about 60%-65% among patients with FTD or unspecified dementia.

**Conclusions:**

Patients with dementia-related psychosis had a high burden of comorbidities, frailty, emergency department visits, infections, and death. Specifically, after DRP diagnosis, patients with DLB and VD had the highest burden of clinical events of interest.

**Supplementary Information:**

The online version contains supplementary material available at 10.1186/s12877-022-03489-3.

## Background

Neuropsychiatric symptoms such as behavioral disturbances and psychosis are common in dementia [1]. The prevalence of dementia-related psychosis—including hallucinations, delusions, and/or formal thought disorder—varies across dementia types, with estimates ranging between 10 and 83% [2–6]. A recent study using the United States (US) Medicare claims database reported that the cumulative incidence of a psychosis diagnosis at 2, 5 and 9 years after dementia was 13.9%, 25.5%, and 30.5%, respectively [7].

Although a substantial proportion of patients with dementia also experience psychosis symptoms, there are no approved treatments specifically for psychosis in patients with dementia, with the exception of pimavanserin, which is approved for the treatment of hallucinations and delusions related to Parkinson’s disease, which may include those with or without dementia [8]. Some classes of drugs, mostly antipsychotics, have been used off-label for the management of psychosis among patients with dementia [6]. However, on the basis of a review of clinical trial data, the US Food and Drug Administration (FDA) determined that the use of antipsychotics in older adults with psychosis and dementia is associated with increased mortality [9, 10]; therefore all antipsychotic drug labeling in the US contains a boxed warning for increased risk of mortality in older patients with psychosis and dementia [11]. Use of atypical antipsychotics is also associated with other risks, including falls and fractures, aspiration pneumonia, infections, and other conditions [12–15], and the American Geriatrics Society Beers Criteria® recommend that antipsychotics be used with caution in older adults [16].

Recent studies have reported that compared with older adults with dementia without psychosis, those with dementia-related psychosis have more serious risks, including falls and fractures, higher healthcare utilization, and higher mortality rates [7, 17]. Specifically, the authors of a prior study conducted using Medicare claims from 2008 to 2016 found that patients with dementia-related psychosis had a hazard ratio of 2.4 (95% confidence interval [CI], 2.3–2.4) to enter in long-term care and a mortality hazard ratio of 2.1 (95% CI, 2.0–2.1) compared with those with dementia without psychosis [7]. Another study of patients with dementia who received commercial insurance or Medicare Advantage with Part D benefits during 2008 to 2018 found that a higher proportion of patients with psychosis experienced falls/fractures (psychosis group, 28%; no psychosis group, 14%) or cardiovascular effects (psychosis group, 7%; no psychosis group, 4%) compared with patients without psychosis [17].

Given that dementia is composed of various dementia types, each with its own underlying etiology and disease trajectory, this current study describes the similarities and differences across the dementia types in the incidence of clinical events and treatment patterns after patients with dementia are diagnosed with psychosis.

## Methods

### Data source

This descriptive assessment used data from US Medicare claims, a federally funded insurance program for adults aged ≥ 65 years and those with certain disabilities or end-stage renal disease. From the Medicare fee-for-service claims database, we obtained information from Part A (hospital insurance), Part B (physician services and outpatient care), and Part D (outpatient prescription drug coverage). Patients with newly diagnosed dementia-related psychosis were identified in the years 2013–2018, and data as far back as 1999 were used to define patient baseline characteristics. We used Medicare claims to identify diagnoses and procedures from inpatient admissions, emergency department (ED) visits, outpatient/physician encounters, home healthcare providers, and durable medical equipment providers; medication dispensings were identified from pharmacies. Information on inpatient oral medication dispensing was not available. All eligible individuals were included.

### Study population

This was an observational (noninterventional), retrospective cohort study using longitudinal, administrative insurance billing claims data for older adults with Medicare coverage. Adult patients aged ≥ 65 years and entitled to Medicare coverage based on age were eligible. Patients were identified at their first recorded psychosis diagnosis between January 1, 2013, and December 31, 2018, and were required to have a previous or concurrent diagnosis of dementia (Alzheimer’s disease [AD], Parkinson’s disease dementia [PDD], dementia with Lewy bodies [DLB], frontotemporal dementia [FTD], vascular dementia [VD], or unspecified dementia) in any setting or diagnostic position and a minimum of 12 months of continuous enrollment in Medicare Parts A, B, and D before the date of the psychosis diagnosis (index date). The *International Classification of Diseases, Ninth Revision, Clinical Modification* (ICD-9-CM) and *International Classification of Diseases, Tenth Revision, Clinical Modification* (ICD-10-CM) codes used to identify psychosis and dementia diagnoses are included in Additional File 1, Tables S-1 through S-4. Antipsychotic medications were not used to define psychosis because of substantial off-label usage of antipsychotics among older individuals, those with dementia, and residents of long-term care facilities without documented psychosis diagnoses [18–20]; additionally, antipsychotics are approved by FDA for the treatment of major depressive disorder and are commonly used for the treatment of other psychiatric disorders, including post-traumatic stress disorder.

The construction of the study cohort is illustrated in Additional File 1, Figure S-1. To identify new-onset psychosis, patients were excluded if they had a psychosis diagnosis or antipsychotic medication at any time before the index date, or a diagnosis of bipolar or schizophrenic disorders at any time on or before the index date. Bipolar and schizophrenic disorders are often treated with antipsychotics, and therefore these patients were excluded to ensure that the study population included those with psychosis related to dementia and not to these other conditions. Patients were followed from the index date until the first occurrence of the following: end of study (December 31, 2018); disenrollment from Medicare Part A, B, or D; diagnosis of a bipolar or schizophrenic condition; or death. Patients were classified to a specific type of dementia at the index date based on diagnosis codes. The final study cohort comprised patients with prevalent dementia and an incident psychosis diagnosis, which defined the cohort entry date (i.e., index date) and start of follow-up.

### Variables of interest

Demographic and clinical characteristics were evaluated on or before the index date. Clinical characteristics were selected to describe conditions that are highly prevalent in patients with dementia and psychosis and that can affect healthcare utilization or quality of life; the assessed clinical characteristics included comorbidities, the Charlson Comorbidity Index (CCI) [21], a frailty index [22, 23], comedications, and healthcare utilization (See Additional File 1, Supplementary Methods). Psychosis symptoms were assessed on the index date by the presence of an ICD-9-CM or ICD-10-CM code for hallucinations and/or delusions, or other unspecified symptoms (Additional File 1, Tables S-1 and S-2); patients with only unspecified psychosis symptom codes (i.e., without a specific diagnosis code for hallucinations and/or delusions) were categorized as having “other, unspecified” symptoms.

During follow-up, antipsychotic treatment status was assessed and categorized as treated, untreated, and died at defined timepoints. Antipsychotic prescriptions were identified using pharmacy dispensing data, and continuous exposure to any antipsychotic medication was estimated using the days’ supply of medication indicated at medication dispensing; we allowed a grace period of up to 50% of the prescription’s day supply for a subsequent prescription to be dispensed for the patient to be considered continuously treated. Events of interest (selected on the basis of the high frequency) that were assessed during follow-up included falls and fractures, aspiration pneumonia, serious hospitalized infections, parenteral anti-infective use, skilled nursing facility admissions, hospital admissions, home healthcare episodes, ED visits, and death (Additional File 1, Supplementary Methods).

### Statistical analysis

Patient characteristics identified before the index date (psychosis diagnosis) were described by dementia type with counts and percentages for categorical variables and means and standard deviations (SDs) for continuous variables.

The following descriptive analyses were conducted by dementia type (AD, PDD, DLB, FTD, VD, and unspecified dementia):

1. The crude incidence rate (IR) of each event of interest was calculated as the number of events occurring during follow-up divided by the total person-years of follow-up; exact 95% CIs were estimated [24]. Multiple events per person were included.
2. Probability of survival over time was plotted with Kaplan–Meier survival plots.
3. To describe the potentially varying incidence of recurrent events over time, the daily mean cumulative count [25] of each event of interest was estimated and plotted as the average number of events experienced by a cohort member at a given time point through follow-up. The mean cumulative count point estimate and 95% CI for the last day of follow-up were calculated via a nonparametric bootstrapping procedure with 100 resamples.
4. Characteristics of initial antipsychotic treatment in and patterns of antipsychotic medication use throughout follow-up were assessed using Sankey diagrams, which display changes in treatment state over time [26].

Analyses were performed using SAS version 9.4 (SAS Institute, Inc.). The RTI International Institutional Review Board (IRB) determined that the study met the criteria for exemption from IRB review and granted a waiver from requiring written informed consent. The US Centers for Medicare and Medicaid Services’ (CMS) Privacy Board approved the use of Medicare Research Identifiable Files for this study under a data use agreement with RTI Health Solutions.

## Results

**Fig. 1:**
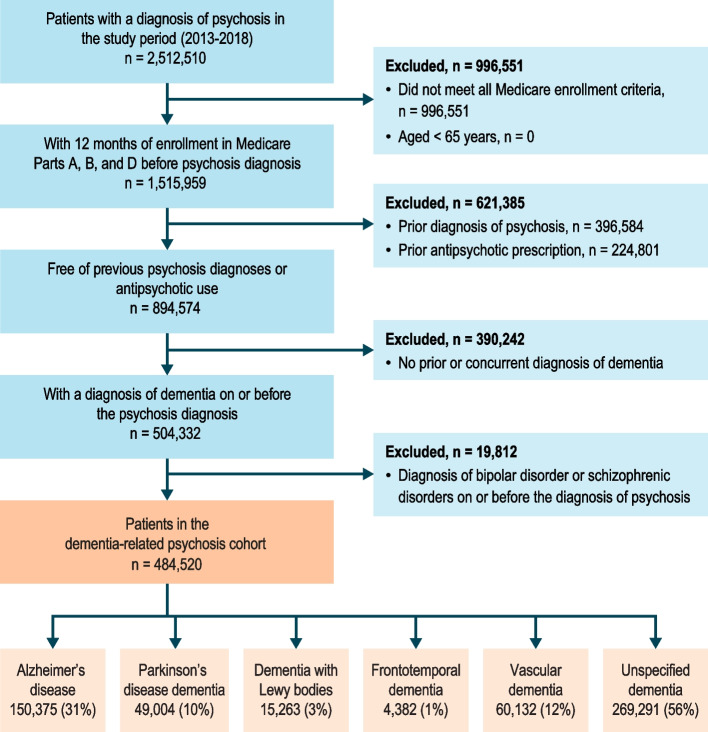
Cohort attrition

A total of 2,512,510 patients diagnosed with psychosis were identified, and 484,520 patients (19.3%) met all eligibility criteria (Fig. 1). Selected patient characteristics by dementia type are shown in Table 1. The mean (SD) age ranged from 81 (7.6) years in patients with FTD to 85 (7.1) years in patients with AD. The majority of patients with AD, FTD, VD, and unspecified dementia were female, while the majority of patients with DLB were male; the proportions of females and males were nearly equal in patients with PDD. In each dementia group, the majority of patients were non-Hispanic white for all dementia types (≥ 80% in each dementia group). Low-income status, as defined by receiving subsidized Medicare Part D coverage, varied across dementia types, with the lowest proportion being among those with FTD (26.4%) and the highest among those with VD (40.4%).

**Table 1:** Baseline selected characteristics of patients with dementia-related psychosis

Characteristic	AD (*n* = 150,375)	PDD (*n* = 49,004)	DLB (*n* = 15,263)	FTD (*n* = 4,382)	VD (*n* = 60,132)	Unspecified dementia^a^ (*n* = 269,291)	All dementia-related psychosis (*N* = 484,520)
Age at DRP diagnosis (index date), n (%)							
Years, mean (SD)	85 (7.1)	82 (7.2)	82 (6.9)	81 (7.6)	84 (7.5)	84 (8.1)	84 (7.8)
65 to < 75 years	15,075 (10.0)	8,143 (16.6)	2,662 (17.4)	1,032 (23.6)	7,829 (13.0)	46,555 (17.3)	73,511 (15.2)
75 to < 85 years	55,884 (37.2)	22,051 (45.0)	7,257 (47.5)	1,933 (44.1)	22,683 (37.7)	97,382 (36.2)	180,518 (37.3)
85 to < 95 years	69,356 (46.1)	17,144 (35.0)	4,971 (32.6)	1,271 (29.0)	25,953 (43.2)	106,972 (39.7)	199,146 (41.1)
≥ 95 years	10,060 (6.7)	1,666 (3.4)	373 (2.4)	146 (3.3)	3,667 (6.1)	18,382 (6.8)	31,345 (6.5)
Sex, n (%)							
Female	102,095 (67.9)	24,146 (49.3)	6,909 (45.3)	2,487 (56.8)	38,325 (63.7)	181,256 (67.3)	319,244 (65.9)
Male	48,280 (32.1)	24,858 (50.7)	8,354 (54.7)	1,895 (43.2)	21,807 (36.3)	88,035 (32.7)	165,276 (34.1)
Race/ethnicity, n (%)							
American Indian/Alaska Native	380 (0.3)	111 (0.2)	33 (0.2)	11 (0.3)	175 (0.3)	1,042 (0.4)	1,592 (0.3)
Asian/Pacific islander	3,575 (2.4)	1,389 (2.8)	365 (2.4)	80 (1.8)	1,222 (2.0)	4,971 (1.8)	9,967 (2.1)
Black	12,786 (8.5)	2,342 (4.8)	872 (5.7)	256 (5.8)	6,752 (11.2)	19,554 (7.3)	37,398 (7.7)
Hispanic	8,344 (5.5)	2,320 (4.7)	679 (4.4)	153 (3.5)	2,944 (4.9)	11,164 (4.1)	22,233 (4.6)
Non-Hispanic White	124,292 (82.7)	42,391 (86.5)	13,170 (86.3)	3,841 (87.7)	48,537 (80.7)	230,685 (85.7)	409,863 (84.6)
Other	686 (0.5)	268 (0.5)	88 (0.6)	22 (0.5)	350 (0.6)	1,182 (0.4)	2,226 (0.5)
Unknown	312 (0.2)	183 (0.4)	56 (0.4)	19 (0.4)	152 (0.3)	693 (0.3)	1,241 (0.3)
Socioeconomic information, n (%)							
Low-income status^b^	54,762 (36.4)	13,943 (28.5)	4,129 (27.1)	1,158 (26.4)	24,280 (40.4)	81,179 (30.1)	156,457 (32.3)
Not low-income status^c^	95,613 (63.6)	35,061 (71.5)	11,134 (72.9)	3,224 (73.6)	35,852 (59.6)	188,112 (69.9)	328,063 (67.7)
Recorded dementia diagnosis occurred before psychosis diagnosis (index date), n (%)							
0 to 182 days	18,563 (12.3)	9,691 (19.8)	2,724 (17.8)	531 (12.1)	10,842 (18.0)	107,591 (40.0)	144,200 (29.8)
183 to 365 days	8,793 (5.8)	3,746 (7.6)	1,011 (6.6)	237 (5.4)	3,949 (6.6)	24,737 (9.2)	39,287 (8.1)
> 365 days	123,019 (81.8)	35,567 (72.6)	11,528 (75.5)	3,614 (82.5)	45,341 (75.4)	136,963 (50.9)	301,033 (62.1)
Previous diagnosis of mild cognitive impairment, yes, n (%)	26,936 (17.9)	9,696 (19.8)	3,627 (23.8)	1,092 (24.9)	10,481 (17.4)	28,441 (10.6)	66,197 (13.7)
Type of psychosis symptomatology^d^ at the index date, n (%)							
Hallucinations	29,859 (19.9)	17,408 (35.5)	5,801 (38.0)	1,042 (23.8)	11,870 (19.7)	59,089 (21.9)	108,793 (22.5)
Delusions	14,757 (9.8)	3,059 (6.2)	1,036 (6.8)	500 (11.4)	6,671 (11.1)	16,097 (6.0)	35,968 (7.4)
Both hallucinations and delusions	1,024 (0.7)	348 (0.7)	165 (1.1)	37 (0.8)	443 (0.7)	1,386 (0.5)	2,921 (0.6)
Other, unspecified^ e^	104,735 (69.6)	28,189 (57.5)	8,261 (54.1)	2,803 (64.0)	41,148 (68.4)	192,719 (71.6)	336,838 (69.5)
Charlson Comorbidity Index score, n (%)							
Mild, scores 1 to 2	13,254 (8.8)	5,108 (10.4)	1,753 (11.5)	547 (12.5)	3,080 (5.1)	34,931 (13.0)	54,382 (11.2)
Moderate, scores 3 to 4	27,728 (18.4)	8,612 (17.6)	2,925 (19.2)	930 (21.2)	8,497 (14.1)	48,897 (18.2)	87,395 (18.0)
Severe, scores ≥ 5	109,393 (72.7)	35,284 (72.0)	10,585 (69.4)	2,905 (66.3)	48,555 (80.7)	185,463 (68.9)	342,743 (70.7)
Frailty index (predicted probability of dependency), n (%)							
Mild, < 5%	5,913 (3.9)	1,149 (2.3)	499 (3.3)	334 (7.6)	2,112 (3.5)	34,183 (12.7)	43,011 (8.9)
Moderate, 5% to < 20%	40,215 (26.7)	9,680 (19.8)	3,093 (20.3)	1,560 (35.6)	12,751 (21.2)	87,922 (32.6)	143,441 (29.6)
Severe, ≥ 20%	104,247 (69.3)	38,175 (77.9)	11,671 (76.5)	2,488 (56.8)	45,269 (75.3)	147,186 (54.7)	298,068 (61.5)
Use of skilled nursing facility services within the 12 months prior to the index date, yes	41,220 (27.4)	14,584 (29.8)	4,523 (29.6)	1,042 (23.8)	21,422 (35.6)	67,236 (25.0)	128,907 (26.6)
Use of home health aide/nurse within the 12 months prior to the index date, yes	55,081 (36.6)	21,380 (43.6)	6,595 (43.2)	1,516 (34.6)	24,050 (40.0)	90,538 (33.6)	171,935 (35.5)

Notes: Although a patient’s psychosis diagnosis was required to occur during the study period (2013–2018), all of a patient’s available Medicare data before their psychosis diagnosis, as far back as 1999, was used to define characteristics. Chronic conditions were evaluated using all of a patient’s available baseline data; short-term conditions, healthcare utilization, and comedications were evaluated in the year before the psychosis diagnosis to characterize the patients at the time of the psychosis diagnosis. Dementia types may not be mutually exclusive. A single patient may have received a diagnosis for more than one specific type of dementia

*AD* Alzheimer’s disease, *DLB* dementia with Lewy bodies, *FTD* frontotemporal dementia, *PDD* Parkinson’s disease dementia, *SD* standard deviation, *VD* vascular dementia

^a^Patients with only unspecified dementia codes without any specific type codes had a value of “yes” for the dementia, unspecified variable

^b^Defined as patients having subsidized Part D coverage

^c^Defined as patients having nonsubsidized Part D coverage

^d^The *International Classification of Diseases, Ninth* and *Tenth Revision, Clinical Modification* codes for defining the psychosis symptomatology are provided in the Additional File 1, Table S-1 and Table S-2

^e^A patient was labeled as having “other, unspecified” symptoms if she or he had only unspecified codes for symptoms without a diagnosis of hallucinations or delusions

For all specified dementia types, more than 70% of patients had a recorded dementia diagnosis more than 365 days before the psychosis diagnosis. Regarding type of psychosis symptomatology at the index date, PDD and DLB had the highest prevalence of hallucinations (35.5% and 38.0%, respectively), and FTD and VD had the highest prevalence of delusions (11% each). However, most patients’ first recorded psychosis diagnosis had unspecified psychosis symptoms.

Overall, patients with dementia and psychosis had a high burden of baseline comorbidities (Table 1 and Additional File 1, Table S-5); between 69% (unspecified dementia) and 81% (VD) had CCI scores indicating severe comorbidity, and between 55% (unspecified dementia) and 78% (PDD) had severe estimated frailty index scores (Table 1). Prior to the psychosis diagnosis, the majority of patients across all dementia types had a previous diagnosis of hypertension and hypertensive heart disease (range, 93.6%-98.1%), hyperlipidemia (range, 93.4%-95.4%), chronic cardiovascular disease (range, 83.5%-92.2%), cerebrovascular disease (range, 69.6%-85.4%), chronic obstructive pulmonary disease (range, 52.6%-62.8%), mood disorders (range, 54.3%-66.9%), and anxiety disorders (range, 54.0%-60.7%) (Additional File 1, Table S-5). In the year prior to the psychosis diagnosis, patients across all dementia types commonly experienced urinary tract infections (range, 41.0%-50.3%), falls and/or fractures (range, 30.4%-38.5%), lower respiratory tract infections (range, 18.1%-25.2%), and serious infections requiring hospitalization (range, 14.9%-21.2%) (Additional File 1, Table S-5). Although the prevalence of all comorbidities were similar across the dementia types, patients with VD had the highest proportions of patients across most measured chronic and acute comorbidities at baseline, with the exception of the following: malignancies (highest in PDD [39.6%]) and osteoporosis (highest in AD [56.2%]), falls and/or fractures (highest in PDD [38.5%] and DLB [38.3%]), upper respiratory infections (highest in unspecified dementia [11.2%]), and aspiration pneumonia (highest in DLB [5.9%]) (Additional File 1, Table S-5).

Comedication use across the dementia types at baseline is presented in Additional File 1, Table S-6. Common medication use included acetylcholinesterase inhibitors (range, 15.0%-58.7%), antidepressants (range, 51.3%-64.6%), antihypertensives (range, 63.9%-76.6%), cholesterol-lowering medications (range, 49.6%-56.8%), and oral anti-infectives (range, 47.3%-51.1%). The use of acetylcholinesterase inhibitors varied between the different dementia types: the prevalence of use was lower in unspecified dementia (15%) and PDD (36%) and higher for all other dementia types (around 50%).

In the year before the psychosis diagnosis, patients in all dementia groups had a mean of 1 hospitalization and 2 ED visits, and patients had a clinic visit on a mean range of 8 (AD) to 11 (PDD) unique days (Additional File 1, Table S-7).

**Fig. 2:**
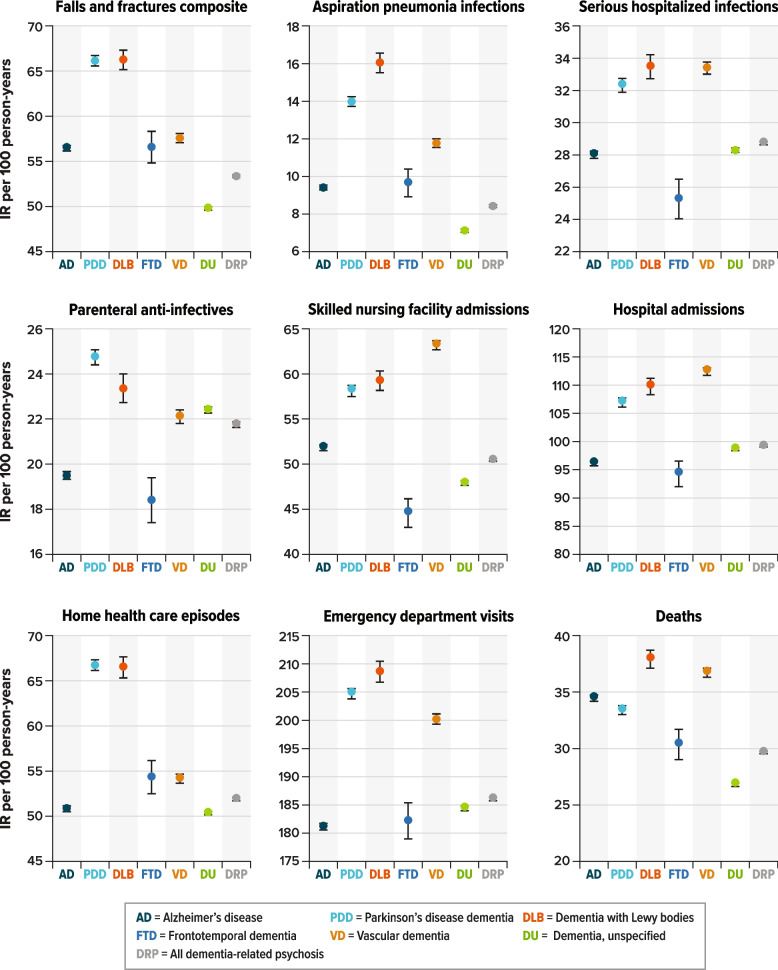
IR plots of events of interest

The IRs of events of interest during follow-up after the psychosis diagnosis by dementia type are presented in Fig. 2 and Additional File 1, Table S-8. Overall, patients with DLB had the highest IRs per 100 person-years (95% CI) for most events of interest, including falls and/or fractures (66.25 [65.20–67.30]), aspiration pneumonia (16.04 [15.52–16.56]), serious hospitalized infections (33.47 [32.71–34.22]), ED visits (208.58 (206.73–210.43]), and death (37.92 [37.13–38.71]). Other dementia types had high IRs for specific events of interest after being diagnosed with psychosis. For example, after their psychosis diagnosis, patients with VD had high IRs (95% CI) per 100 person-years for skilled nursing facility admissions (63.13 [62.59–63.67]), hospital admissions (112.37 [111.66–113.07]), and death (36.78 [36.38–37.18]). Parkinson’s disease dementia had a high IR for falls and fractures (IR = 66.16 per 100 person-years; 95% CI, 65.59–66.73). Alzheimer’s disease, FTD, and unspecified dementia had the lowest IR for almost all events of interest, particularly for aspiration pneumonia infections and ED visits. Finally, the highest mortality rate (95% CI) per 100 person-years was in patients with DLB (37.92 [37.13–38.71]), followed by patients with VD (36.78 [36.38–37.18]) and patients with AD (34.51 [34.27–34.74]).

The mean cumulative counts for each event of interest during follow-up after the psychosis diagnosis are presented in Additional File 1, Figure S-2. Over 6 years of follow-up after the psychosis diagnosis, the mean cumulative counts for falls and/or fractures ranged from 1.2 for patients with VD to 1.5 for patients with PDD. For skilled nursing facility stays, the 6-year mean cumulative counts ranged from 38.7 days for FTD to 51.1 days for VD. Finally, for ED visits, the 6-year mean cumulative count ranged from 3.8 days for AD to 4.7 days for unspecified dementia.

**Fig. 3:**
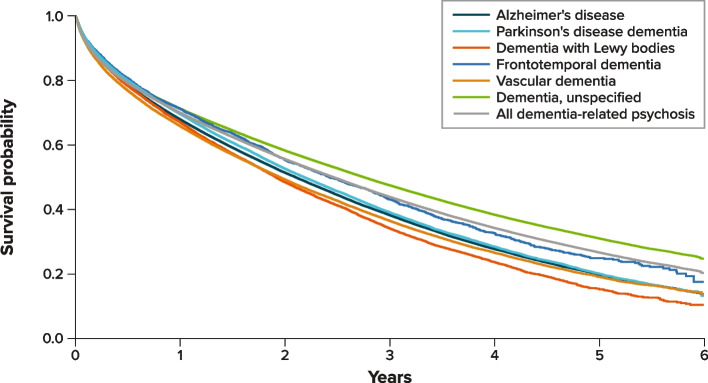
KM curves

The cumulative probability of death after the psychosis diagnosis by dementia type is presented in Fig. 3. One year after the psychosis diagnosis, approximately 30% of patients had died in all dementia types. After 5 years of follow-up, death occurred in approximately 80% of patients with DLB, VD, AD, or PDD, and in approximately 60%-65% of patients with FTD or unspecified dementia.

Characteristics of initial antipsychotic treatment are presented in Additional File 1, Table S-9. The mean number of days of treatment initiation with antipsychotics after the index date (i.e., date of psychosis diagnosis) ranged from approximately 180 days in patients with DLB, FTD, and VD to 278 days in patients with unspecified dementia. The most commonly prescribed antipsychotic drug during follow-up among all dementia types was quetiapine. Antipsychotic treatment status by dementia type is presented in Additional File 1, Figure S-3. Six months after the diagnosis of psychosis, the proportion of patients initiating treatment with antipsychotics ranged from 7% for unspecified dementia to 14% for DLB and FTD. Among all dementia types, the percentage of patients treated with antipsychotics decreased over follow-up.

## Discussion

This study showed that patients with dementia-related psychosis (those with a psychosis diagnosis at the start of follow-up) had a high burden of comorbidities and complications after their psychosis diagnosis, but this burden varied by dementia type. Patients with DLB had the highest IR for most events of interest after their psychosis diagnosis compared to patients with other types of dementia. In addition, patients with VD had higher IRs for healthcare utilization, including skilled nursing facility admissions and hospital admissions. The cumulative probability of death after 5 years of the psychosis diagnosis was higher among patients with DLB, VD, AD, or PDD (around 80%) than in patients with FTD or unspecified dementia (60%-65%). Among all dementia types, the percentage of patients initiating treatment with antipsychotics was low, with a high rate of discontinuation over follow-up.

The most common adverse health events during follow-up, with ≥ 100 events per 100 person-years, were ED visits and hospital admissions. Our results were similar to those reported in a retrospective study using commercial or Medicare Advantage insurance data in 2010–2018 [17], which reported 12-month cumulative incidences for ≥ 1 outpatient visit, ≥ 1 ED visit, and ≥ 1 inpatient stay to be 80%, 65%, and 47%, respectively. In another study of patients with dementia and psychosis conducted in Medicare [7], 16% of patients had entered long-term care within 2 years of dementia-related psychosis diagnosis.

In the current study, we observed an overall high mortality rate (95% CI) per 100 person-years ranging from 37.92 (37.13–38.71) among patients with DLB to 26.78 (26.64–26.92) among patients with unspecified dementia. Our results were similar to that observed by Wetmore et al. [7], which reported that the cumulative incidence of death at 1 year after patients with dementia were diagnosed with psychosis was approximately 35%, at 3 years 65%, and at 5 years 80%.

The American Psychiatric Association recommends that only severe cases of dementia and psychosis should be treated with antipsychotics [27], and we observed that, at 6 months after dementia-related psychosis diagnosis, the percentage of patients treated with an antipsychotic ranged from 7% for dementia unspecified to 14% for patients with psychosis and DLB or FTD, with quetiapine being the most commonly prescribed; however, the percentage of patients under treatment with antipsychotics decreased over follow-up. The observed patterns of antipsychotic use in our study appear generally consistent with guidelines that advise providers to avoid the use of haloperidol when managing these symptoms in patients with dementia-related psychosis. Our study required patients to be free of antipsychotic use before dementia-related psychosis diagnosis—a large number of patients were excluded from the study for having antipsychotic use before receiving a psychosis diagnosis (Fig. 1)—and many patients may begin antipsychotic use before receiving a formal psychosis diagnosis. Additionally, our results showed that the use of acetylcholinesterase inhibitors and antidepressants were common in patients with dementia-related psychosis. In a study of US veterans with dementia, the 10-year cumulative incidence of antipsychotic use was 51.5% [28]. In a UK study, 30% of patients with a dementia and “psychosis symptoms and no agitation” were treated with antipsychotics [29]. A review including studies published to 2014 reported a pooled prevalence of antipsychotic treatment among patients with dementia of 27.5%, with a higher prevalence among patients in long-term care [30].

This study has several strengths, including a large sample size of more than 484,520 patients with a diagnosis of dementia-related psychosis and the assessment of a number of clinical events of interest by type of dementia. However, the results presented in the current study should be evaluated in light of potential limitations. Given the nature of the underlying US Medicare population, our study is generalizable only to older adults (aged 65 years and older) residing in the US and receiving Medicare fee-for-service benefits. Among our inclusion criteria, we required patient enrollment in Medicare Parts A, B, and D, which might result in a slightly more selected population, as Part D is an optional program. Identification of some characteristics (e.g., psychosis symptoms, dementia type and stage, cause of death) is challenging in an automated healthcare database using coded diagnoses on billing claims that are created for reimbursement rather than for clinical or research purposes (e.g., the most common dementia codes were for “unspecified dementia,” consistent with prior studies conducted in Medicare [7, 31], and the most common psychosis diagnoses were for general psychosis rather than specifically for hallucinations or delusions, potentially because of the absence of comprehensive clinical assessments or treatment by nonspecialists). Moreover, the list of ICD-9-CM and ICD-10-CM codes to identify psychosis and dementia could not be validated or compared with clinical charts. Only psychosis in different types of dementia was identified in the current study. Other impairing behavioral symptoms in different types and stages of dementia, such as agitation/aggression, depression, anxiety, and apathy, were not included in our assessment. The dates of claims represent the dates of healthcare encounters resulting in a bill; thus, use of claims may result in misclassification of disease or symptom onset. Prescription claims may not reflect actual medication exposure, as only pharmacy dispensing information is captured, not actual patient use. Some patients with dementia and psychosis may not have been captured with our study population definition, which first required a claim for dementia followed by a claim for psychosis. The inclusion of patients having a psychosis diagnosis and a subsequent code for dementia would introduce immortal person-time given that the index date is the date of the psychosis diagnosis. To avoid introducing immortal person-time bias, we restricted our population to patients with a code for dementia and a concurrent or subsequent code for psychosis.

## Conclusion

The results from this study are intended to describe the characteristics and burdens of patients with dementia-related psychosis rather than to estimate causal associations between dementia-related psychosis and any outcomes. In this descriptive study, we observed that dementia-related psychosis is a complex disorder involving both neurological and psychiatric symptoms and that those patients additionally experience a substantial burden of non-neuropsychiatric events after diagnosis, with high rates of ED visits, hospital admissions, infections, anti-infective use, and death. Among the different dementia types, patients with DLB and VD showed the highest burden of nonpsychiatric symptoms.

## Supplementary Information

**Additional file 1.** 

## Data Availability

The CMS Privacy Board approved access to Medicare Research Identifiable Files for use in this study under a data use agreement with RTI Health Solutions, under which only those listed on the data use agreement can access the study-specific data. Data can be requested for use from CMS by establishing a data use agreement with CMS (https://resdac.org/).

## Abbreviations

AD: Alzheimer’s disease
CCI: Charlson Comorbidity Index
CI: Confidence interval
CMS: Centers for Medicare and Medicaid Services
DLB: Dementia with Lewy bodies
ED: Emergency department
FTD: Frontotemporal dementia
IR: Incidence rate
IRB: Institutional review board
PDD: Parkinson’s disease dementia
SD: Standard deviation
US: United States
VD: Vascular dementia
