# Supplementary material for: Clinical outcomes and treatment patterns of older adults with dementia-related psychosis by dementia type in the United States
Source: BMC Geriatr. 2022 Oct 6;22:784. doi: 10.1186/s12877-022-03489-3 (PMC9541053; doi:10.1186/s12877-022-03489-3)
Supplement: Supplementary file 1 — Additional file 1. [file 12877_2022_3489_MOESM1_ESM.docx]

Supporting Information

Contents

[1 Supplementary Methods: Study Design, Codes to Identify Psychosis and Dementia, and Operational Definitions of Baseline Characteristics and Events of Interest 3](#_Toc114047835)

[1.1 Study Design 3](#_Toc114047836)

[1.2 Codes to Identify Psychosis 5](#_Toc114047837)

[1.3 Codes to Identify Dementia 7](#_Toc114047838)

[1.4 Description of Comorbidities, Comedications and Healthcare Utilization 11](#_Toc114047839)

[1.4.1 Comorbidities 11](#_Toc114047840)

[1.4.2 Comedications 12](#_Toc114047841)

[1.4.3 Healthcare Utilization 13](#_Toc114047842)

[1.5 Operational Definition of Falls and Fractures 14](#_Toc114047843)

[1.6 Operational Definition of Infections 16](#_Toc114047844)

[1.6.1 Operational Definition of Aspiration Pneumonia 16](#_Toc114047845)

[1.6.2 Operational Definition of Serious Hospitalized Infection 16](#_Toc114047846)

[1.7 Operational Definition of Healthcare Utilization 16](#_Toc114047847)

[1.7.1 Operational Definition of Skilled Nursing Facility Services 16](#_Toc114047848)

[1.7.2 Operational Definition of Home Healthcare Episodes 16](#_Toc114047849)

[1.7.3 Operational Definition of Hospitalizations 17](#_Toc114047850)

[1.7.4 Operational Definition of Hospitalizations 17](#_Toc114047851)

[1.7.5 Operational Definition of Parenteral Anti-infective Use 17](#_Toc114047852)

[1.8 Operational Definition of Death 17](#_Toc114047853)

[2 Results Tables and Figures 18](#_Toc114047854)

[3 References 43](#_Toc114047855)

Tables

[Table S-1. Diagnosis Codes for Identifying Psychosis: ICD-9-CM 5](#_Toc114047700)

[Table S-2. Diagnosis Codes for Identifying Psychosis: ICD-10-CM 6](#_Toc114047701)

[Table S-3. Diagnosis Codes for Identifying Dementia: ICD-9-CM 7](#_Toc114047702)

[Table S-4. Diagnosis Codes for Identifying Dementia: ICD-10-CM 9](#_Toc114047703)

[Table S-5. Comorbidities of Patients With Dementia-Related Psychosis 18](#_Toc114047704)

[Table S-6. Comedication Use Among Patients With Dementia-Related Psychosis 20](#_Toc114047705)

[Table S-7. Healthcare Utilization in Patients With Dementia-Related Psychosis Assessed in the Year Before and Not Including the Index Date 21](#_Toc114047706)

[Table S-8. Incidence Rates of Events of Interest in Patients With Dementia-Related Psychosis 22](#_Toc114047707)

[Table S-9. Characteristics of Initial Antipsychotic Treatment in Patients With Dementia-Related Psychosis 34](#_Toc114047708)

Figures

[Figure S-1. Variable Assessment Windows Relative to the Study Index Date 3](#_Toc114047709)

[Figure S-2. Mean Cumulative Counts for Each Event of Interest in Patients With Dementia-Related Psychosis 26](#_Toc114047710)

[Figure S-3. Antipsychotic Treatment Status Over Follow-up in Patients With Dementia-Related Psychosis 36](#_Toc114047711)

# Supplementary Methods: Study Design, Codes to Identify Psychosis and Dementia, and Operational Definitions of Baseline Characteristics and Events of Interest

## Study Design

1. Variable Assessment Windows Relative to the Study Index Date

DRP = dementia-related psychosis; HCU = healthcare utilization.

Note: figure template available at [www.repeatinitiative.org](http://www.repeatinitiative.org)

^a^ Chronic cardiovascular disease, cerebrovascular diseases, hypertension, diabetes mellitus (type 1 or 2), hyperlipidemia, malignancies, chronic kidney disease and other renal disorders, liver disease, chronic obstructive pulmonary disease, osteoporosis, psychiatric disorders, Charlson Comorbidity Index score, acetylcholinesterase inhibitors, antidepressants, anxiolytics, sedatives, drugs used in opioid dependence.

^b^ Use of skilled nursing facility; use of home healthcare services; cardiovascular events; diseases of arteries, arterioles, and capillaries; falls and fractures; aspiration pneumonia; upper respiratory tract infections; lower respiratory tract infections; urinary tract infections; severe sepsis; serious hospitalized infections; overall frailty index; antihypertensives; cholesterol-lowering medications; anticoagulants; treatments for Parkinson’s disease; anti-inflammatory drugs; bronchodilators; osteoporosis treatments; oral anti-infectives; parenteral anti-infectives; hospitalizations; emergency department visits; clinic visits.

^c^ End of study (December 31, 2018); disenrollment from Medicare Part A, B, or D; diagnosis of a bipolar or schizophrenic condition; or death.

## Codes to Identify Psychosis

1. Diagnosis Codes for Identifying Psychosis: ICD-9-CM

| Code | Description |
| --- | --- |
| **Hallucinations** | |
| 293.82 | Psychotic disorder with hallucinations in conditions classified elsewhere |
| 368.16 | Psychophysical visual disturbances |
| 780.1 | Hallucinations |
| **Delusions** | |
| 293.81 | Psychotic disorder with delusions in conditions classified elsewhere |
| 297.1 | Delusional disorder |
| 297.2 | Paraphrenia |
| **Other** | |
| 297.0 | Paranoid state, simple |
| 297.3 | Shared psychotic disorder |
| 297.8 | Other specified paranoid states |
| 297.9 | Unspecified paranoid state |
| 298.0 | Depressive type psychosis |
| 298.1 | Excitative type psychosis |
| 298.2 | Reactive confusion |
| 298.3 | Acute paranoid reaction |
| 298.4 | Psychogenic paranoid psychosis |
| 298.8 | Other and unspecified reactive psychosis |
| 298.9 | Unspecified psychosis |

ICD-9-CM = *International Classification of Diseases, Ninth Revision, Clinical Modification*.

1. Diagnosis Codes for Identifying Psychosis: ICD-10-CM

| Code | Description |
| --- | --- |
| **Hallucinations** | |
| F06.0 | Psychotic disorder with hallucinations due to known physiological condition |
| H53.16 | Psychophysical visual disturbances |
| R44.0 | Auditory hallucinations |
| R44.1 | Visual hallucinations |
| R44.2 | Other hallucinations |
| R44.3 | Hallucinations, unspecified |
| R48.3 | Visual agnosia |
| **Delusions** | |
| F06.2 | Psychotic disorder with delusions due to known physiological condition |
| F22 | Delusional disorders |
| **Other** | |
| F23 | Brief psychotic disorder |
| F24 | Shared psychotic disorder |
| F28 | Other psychotic disorder not due to a substance or known physiological condition |
| F29 | Unspecified psychosis not due to a substance or known physiological condition |
| F32.3 | Major depressive disorder, single episode, severe with psychotic features |
| F33.3 | Major depressive disorder, recurrent, severe with psychotic symptoms |
| F44.89 | Other dissociative and conversion disorders |

ICD-10-CM = *International Classification of Diseases, Tenth Revision, Clinical Modification*.

## Codes to Identify Dementia

1. Diagnosis Codes for Identifying Dementia: ICD-9-CM

| Code | Description |
| --- | --- |
| Alzheimer’s disease | |
| 331.0 | Alzheimer’s disease |
| Vascular dementia | |
| 290.40 | Vascular dementia, uncomplicated |
| 290.41 | Vascular dementia, with delirium |
| 290.42 | Vascular dementia, with delusions |
| 290.43 | Vascular dementia, with depressed mood |
| Dementia with Lewy bodies | |
| 331.82 | Dementia with Lewy bodies |
| Frontotemporal dementia | |
| 331.11 | Pick’s disease |
| 331.19 | Other frontotemporal dementia |
| Parkinson’s disease dementia | |
| 332.0 + Dementia code | Paralysis agitans |
| Dementia not otherwise specified | |
| 290.0 | Senile dementia, uncomplicated |
| 290.10 | Presenile dementia, uncomplicated |
| 290.11 | Presenile dementia with delirium |
| 290.12 | Presenile dementia with delusional features |
| 290.13 | Presenile dementia with depressive features |
| 290.20 | Senile dementia with delusional features |
| 290.21 | Senile dementia with depressive features |
| 290.3 | Senile dementia with delirium |
| 290.9 | Unspecified senile psychotic condition |
| 294.0 | Amnestic disorder in conditions classified elsewhere |
| 294.10 | Dementia in conditions classified elsewhere without behavioral disturbance |
| 294.11 | Dementia in conditions classified elsewhere with behavioral disturbance |
| 294.20 | Dementia, unspecified, without behavioral disturbance |
| 294.21 | Dementia, unspecified, with behavioral disturbance |
| 294.8 | Other persistent mental disorders due to conditions classified elsewhere |
| 294.9 | Unspecified persistent mental disorders due to conditions classified elsewhere |
| 331.2 | Senile degeneration of brain |
| 331.6 | Corticobasal degeneration |
| 331.7 | Cerebral degeneration in diseases classified elsewhere |
| 780.93 | Memory loss |
| 797 | Senility without mention of psychosis |

ICD-9-CM = *International Classification of Diseases, Ninth Revision, Clinical Modification*.

1. Diagnosis Codes for Identifying Dementia: ICD-10-CM

| Code | Description |
| --- | --- |
| Alzheimer’s disease | |
| G30.0 | Alzheimer’s disease with early onset |
| G30.1 | Alzheimer’s disease with late onset |
| G30.8 | Other Alzheimer’s disease |
| G30.9 | Alzheimer’s disease, unspecified |
| Vascular dementia | |
| F01.5 | Vascular dementia |
| F01.50 | Vascular dementia without behavioral disturbance |
| F01.51 | Vascular dementia with behavioral disturbance |
| Dementia with Lewy bodies | |
| G31.83 | Dementia with Lewy bodies |
| Frontotemporal dementia | |
| G31.01 | Pick’s disease |
| G31.09 | Other frontotemporal dementia |
| Parkinson’s disease dementia | |
| G20 + Dementia code | Paralysis agitans |
| Dementia not otherwise specified | |
| F02.80 | Dementia in other diseases classified elsewhere without behavioral disturbance |
| F02.81 | Dementia in other diseases classified elsewhere with behavioral disturbance |
| F03.90 | Unspecified dementia without behavioral disturbance |
| F03.91 | Unspecified dementia with behavioral disturbance |
| F04 | Amnestic disorder due to known physiological condition |
| F02.80 | Dementia in other diseases classified elsewhere without behavioral disturbance |
| F02.81 | Dementia in other diseases classified elsewhere with behavioral disturbance |
| F03.90 | Unspecified dementia without behavioral disturbance |
| F03.91 | Unspecified dementia with behavioral disturbance |
| G31.1 | Senile degeneration of brain, not elsewhere classified |
| G31.85 | Corticobasal degeneration |
| G31.89 | Other specified degenerative diseases of nervous system |
| G31.9 | Degenerative disease of nervous system, unspecified |
| R41.1 | Anterograde amnesia |
| R41.2 | Retrograde amnesia |
| R41.3 | Other amnesia |
| R41.81 | Age-related cognitive decline |
| R54 | Age-related physical debility |

ICD-10-CM = *International Classification of Diseases, Tenth Revision, Clinical Modification*.

## Description of Comorbidities, Comedications and Healthcare Utilization

### Comorbidities

Comorbidities were characterized before and not including the index date using recorded diagnosis codes from inpatient, outpatient, physician, and home healthcare billing claims. For patients in the antipsychotic treatment subcohort, comorbidities were re-evaluated before the antipsychotic treatment initiation date. A binary (present/not present) indicator of the presence of any diagnosis codes for the following conditions occurring during the indicated assessment windows will be created for each variable:

- Assessed at any time before and not including the index date (or antipsychotic treatment initiation date)
- Chronic cardiovascular diseases: conduction disorders, arrhythmias, heart failure
- Cerebrovascular diseases: cerebral hemorrhage (subarachnoid, intracerebral, other nontraumatic), cerebral infarction and stroke, transient ischemic attack, other cerebrovascular disease, and sequelae of cerebrovascular disease
- Hypertension
- Diabetes mellitus (type 1 or 2)
- Hyperlipidemia
- Malignancies
- Chronic kidney disease and other renal disorders
- Liver disease
- Chronic obstructive pulmonary disease
- Osteoporosis
- Mood disorders
- Anxiety disorders
- Other psychiatric disorders: eating disorders, substance abuse disorders, unspecified mental/psychiatric disorders
- Assessed during the 12 months before and not including the index date (or the antipsychotic treatment initiation date)
- Cardiovascular events: ischemic heart disease (including angina pectoris, acute myocardial infarction, other acute or subacute ischemic heart disease, chronic ischemic heart disease), cardiac arrest
- Diseases of arteries, arterioles, and capillaries (including peripheral arterial disease)
- Composite falls and fractures
- Aspiration pneumonia
- Upper respiratory tract infections
- Lower respiratory tract infections
- Urinary tract infections
- Severe sepsis (hospitalized only)
- Serious infection (hospitalized only)

Additionally, the Charlson Comorbidity Index (CCI) [1], and a frailty index [2,3], were estimated.

### Comedications

Pharmacy dispensing and procedure coding records before and not including the index date or antipsychotic treatment initiation date were used to characterize comedications. For patients in the antipsychotic treatment subcohort, comedications were re-evaluated before the antipsychotic treatment initiation date. A binary (present/not present) indicator of the presence of any dispensing codes for the following medications occurring during the indicated assessment windows was created for each medication class:

- Assessed at any time before and not including the index date (or antipsychotic treatment initiation date) in order to identify any history of psychiatric or neurological disorders
- Acetylcholinesterase inhibitors
- Antidepressants
- Anxiolytics
- Sedatives
- Drugs used in opioid dependence
- Assessed during the 12 months before and not including the index date (or the antipsychotic treatment initiation date) in order to identify more recent comorbid conditions
- Antihypertensives
- Cholesterol-lowering medications
- Anticoagulants
- Treatments for Parkinson’s disease
- Anti-inflammatory drugs
- Bronchodilators
- Osteoporosis treatments
- Oral anti-infectives (including pharmacy-dispensed antibiotics, antifungals, and antivirals)
- Parenteral anti-infectives (including outpatient or inpatient injection or intravenous antibiotics, antifungals, and antivirals identified from pharmacy dispensing or procedure coding

### Healthcare Utilization

Healthcare utilization indicators were assessed during the 12 months before and not including the index date. For patients in the antipsychotic treatment subcohort, healthcare utilization was evaluated in the 12 months before and not including the antipsychotic treatment initiation date.

Healthcare utilization indicators of interest included the following:

- Hospitalizations, defined as the number of unique hospital admissions evaluated in the Medicare MedPAR files
- Emergency department visits, identified as the number of unique days with an emergency department admission from the Medicare MedPAR files where the emergency room charge amount was greater than $0 or in the Outpatient file in which the revenue center code indicated an emergency department visit
- Number of unique days during which a clinic visit occurred, evaluated using outpatient and physician billing claims

## Operational Definition of Falls and Fractures

All falls and fractures diagnosed for each person were identified during follow-up. Multiple events per patient were included in analyses. Falls and fractures were evaluated both together as a composite outcome and separately as distinct outcomes.

Falls were identified by ICD-9-CM^[[1]](#footnote-1)^ or ICD-10-CM^[[2]](#footnote-2)^ diagnosis codes for falls occurring in any diagnosis position in Medicare MedPAR, outpatient, or physician billing claims. Repeated codes for falls occurring within the 7 days after and not including the date of an identified fall were assumed to be part of the same event and were not considered separately.

Falls were evaluated in a patient up to 7 days before the index date to ensure that falls occurring after the index date were new events. Falls were identified in the 12 months before the index date as a descriptive comorbidity.

Fractures were identified by diagnosis codes from inpatient, outpatient, or physician billing claims for a fracture (occurring in any diagnosis position) at a specific anatomic site with a procedure code (ICD-9-CM procedure, ICD‑10‑PCS^[[3]](#footnote-3)^, or CPT^[[4]](#footnote-4)^) for a fracture repair occurring in a window from 7 days before to 7 days after the date of the fracture diagnosis (days [−7, 7]). The earlier date of the fracture diagnosis or repair was considered the event date. Each fracture diagnosis and repair code were categorized by anatomic site (skull, vertebrae, trunk, upper limb, hand/wrist/finger, pelvis, hip, femur, lower leg, foot/ankle/toe). Procedure codes for a fracture repair at an unspecified site were included in the procedure code lists for all other sites and could be paired with any site-specific fracture diagnosis. If unspecified fracture diagnosis codes were identified, they were paired with a site-specific repair procedure code for the site to be determined.

All fractures at the same site within the period from “fracture date + 1 day” to “fracture date + 365 days” were considered part of the same event.

Fractures were not reported as individual, site-specific events; only overall fractures were reported. However, after the occurrence of a site-specific fracture, the patient was not at risk to experience a fracture at the same site within the following 12 months. Fractures at *different* sites were considered distinct events even if occurring within the same year; however, additional claims for fractures at multiple sites were considered as one event if the claims occurred within a 7-day window following and not including the day of the initial fracture. The earliest date denoting a fracture within the 8-day period (the date of the first claim and the following 7 days) was considered the date of occurrence in the analyses of fractures.

The 12 months before, but not including, the index date were evaluated for fracture events to ensure identification of new-onset events after the index date and define recurrent site-specific fractures; fractures in the 12 months before the index date were also used as descriptive comorbidities.

Falls and overall fracture occurrences were also evaluated as one composite outcome. All individual fall and fracture events were considered in the analysis. However, if a fall and a fracture event occurred within 7 days of each other (days [0, 7]), they were considered part of the same outcome event for the composite outcome only. The earliest date denoting a fall or fracture within the 8-day period was considered the date of occurrence in the composite falls/fractures analyses.

## Operational Definition of Infections

### Operational Definition of Aspiration Pneumonia

Aspiration pneumonia was defined as a diagnosis code for aspiration pneumonia on an inpatient, emergency department, outpatient, or physician claim. Aspiration pneumonia codes were specific to pneumonia that is attributed to inhalation of food or vomit and is thus separate from codes used to indicate the presence of bacterial or viral pneumonia [4]. In a validation study in Medicare beneficiaries in California, when compared with clinical evidence and physician notes, this singular diagnosis code had a positive predictive value (PPV) of 90.6% [5].

### Operational Definition of Serious Hospitalized Infection

A broader definition of “serious hospitalized infection” was identified as an inpatient diagnosis of meningitis, encephalitis, cellulitis, endocarditis, pneumonia, pyelonephritis, septic arthritis, osteomyelitis, bacteremia, tuberculosis, atypical mycobacteria, cryptococcosis, or aspergillosis. Serious hospitalized infections may have overlapped with the specific infection subtypes described previously. Previous validation studies of these definitions of serious hospitalized infections have demonstrated high PPVs [6,7].

## Operational Definition of Healthcare Utilization

### Operational Definition of Skilled Nursing Facility Services

Utilization of skilled nursing facility services was identified from the Medicare MedPAR files that had an indicator of a skilled nursing facility stay. Admissions were assessed in both of the following forms:

- The number of unique, skilled nursing facility admissions
- The number of days during follow-up spent in a skilled nursing facility

### Operational Definition of Home Healthcare Episodes

Home healthcare episodes were identified from the Medicare Home Health billing claims. Home healthcare was assessed through both of the following forms:

- The number of unique home healthcare episodes
- The number of days during follow-up with a home healthcare episode

### Operational Definition of Hospitalizations

Hospitalizations were identified as the number of unique days with an inpatient admission, identified from inpatient events in the Medicare MedPAR files

- The number of unique inpatient hospitalization admissions
- The number of days during follow-up spent in an inpatient hospitalization

### Operational Definition of Hospitalizations

- Emergency department visits were identified as the number of unique days with an emergency department visit from the Medicare MedPAR files where the emergency room charge amount was greater than $0 or in the Outpatient file where the revenue center code indicated an emergency department visit

### Operational Definition of Parenteral Anti-infective Use

Receipt of injectable or intravenous anti-infectives was also considered. Parenteral anti-infective use was considered as a count of the number of outpatient or inpatient intravenous agent treatment episodes administered or the count of pharmacy-dispensed prescriptions for injectable or intravenous anti-infectives identified in the Part D pharmacy claims during the appropriate follow-up window specified in the study design. Parenteral procedures on consecutive days were considered part of the same treatment episode. Pharmacy dispensing of parenteral dosage forms was also identified in Part D pharmacy claims for at-home administration.

## Operational Definition of Death

A patient’s date of death was identified in Medicare enrollment information.

# Results Tables and Figures

1. Comorbidities of Patients With Dementia-Related Psychosis

| Comorbidity | AD,  n = 150,375 | PDD,  n = 49,004 | DLB,  n = 15,263 | FTD,  n = 4,382 | VD,  n = 60,132 | Unspecified dementia,  n = 269,291 | All dementia-related psychosis,  N = 484,520 |
| --- | --- | --- | --- | --- | --- | --- | --- |
| Assessed at any point before and not including the index date, % |  |  |  |  |  |  |  |
| Chronic cardiovascular disease | 88.6 | 88.9 | 87.5 | 83.5 | 92.2 | 88.0 | 88.4 |
| Cerebrovascular disease | 75.1 | 77.1 | 75.3 | 75.2 | 85.4 | 69.6 | 72.5 |
| Hypertension and hypertensive heart disease | 96.5 | 95.7 | 94.9 | 93.6 | 98.1 | 96.3 | 96.4 |
| Diabetes | 33.1 | 34.9 | 31.8 | 32.5 | 40.7 | 33.9 | 34.1 |
| Hyperlipidemia | 94.3 | 94.3 | 93.4 | 93.7 | 95.4 | 93.5 | 93.9 |
| Malignancies | 36.7 | 39.6 | 38.0 | 36.2 | 37.4 | 37.2 | 37.0 |
| Chronic kidney disease and other renal disorders | 42.8 | 42.5 | 40.3 | 36.1 | 49.3 | 43.6 | 43.6 |
| Liver disease | 24.1 | 25.5 | 23.8 | 24.1 | 25.7 | 25.0 | 24.7 |
| Chronic obstructive pulmonary disease | 59.0 | 57.3 | 54.0 | 52.6 | 62.8 | 61.2 | 60.2 |
| Osteoporosis | 56.2 | 49.0 | 45.3 | 48.2 | 53.1 | 51.7 | 52.6 |
| Mood disorders | 62.1 | 64.4 | 62.8 | 64.0 | 66.9 | 54.3 | 57.8 |
| Anxiety disorders | 57.3 | 59.1 | 56.3 | 59.1 | 60.7 | 54.0 | 55.6 |
| Other psychiatric disorders (eating disorders, substance abuse, or unspecified mental/psychiatric disorders) | 21.8 | 20.9 | 19.7 | 24.2 | 26.2 | 24.5 | 23.6 |
| Assessed during the year before and not including the index date, % |  |  |  |  |  |  |  |
| Cardiovascular events |  |  |  |  |  |  |  |
| Ischemic heart disease | 10.3 | 11.3 | 10.7 | 9.8 | 13.4 | 12.6 | 11.9 |
| Diseases of arteries, arterioles, and capillaries (including peripheral arterial disease) | 41.0 | 42.3 | 40.5 | 38.3 | 47.6 | 38.7 | 40.1 |
| Falls and/or fractures | 33.6 | 38.5 | 38.3 | 31.4 | 36.0 | 30.4 | 32.2 |
| Aspiration pneumonia | 3.7 | 5.3 | 5.9 | 3.8 | 5.2 | 3.4 | 3.7 |
| Infections |  |  |  |  |  |  |  |
| Upper respiratory tract infections | 10.6 | 10.9 | 9.9 | 10.5 | 10.9 | 11.2 | 10.9 |
| Lower respiratory tract infections | 21.0 | 22.2 | 21.5 | 18.1 | 25.2 | 22.6 | 22.2 |
| Urinary tract infections | 46.8 | 46.8 | 46.7 | 41.7 | 50.3 | 41.0 | 43.5 |
| Severe sepsis | 5.7 | 6.3 | 6.6 | 4.7 | 7.5 | 6.2 | 6.1 |
| Serious, hospitalized infections | 16.9 | 18.6 | 18.6 | 14.9 | 21.2 | 18.6 | 18.3 |

AD = Alzheimer’s disease; DLB = dementia with Lewy bodies; FTD = frontotemporal dementia; PDD = Parkinson’s disease dementia; VD = vascular dementia.

1. Comedication Use Among Patients With Dementia-Related Psychosis

| Comedication | AD,  n = 150,375 | PDD,  n = 49,004 | DLB,  n = 15,263 | FTD,  n = 4,382 | VD,  n = 60,132 | Unspecified dementia,  n = 269,291 | All dementia-related psychosis,  N = 484,520 |
| --- | --- | --- | --- | --- | --- | --- | --- |
| Assessed at any point before and not including the index date, % |  |  |  |  |  |  |  |
| Acetylcholinesterase inhibitors | 58.7 | 36.2 | 50.2 | 54.2 | 43.6 | 15.0 | 29.9 |
| Antidepressants | 60.2 | 62.3 | 61.7 | 64.6 | 62.4 | 51.3 | 55.1 |
| Anxiolytics | 10.9 | 10.4 | 9.0 | 10.0 | 11.2 | 10.1 | 10.4 |
| Sedatives | 15.6 | 18.7 | 16.3 | 15.7 | 16.3 | 17.2 | 16.7 |
| Drugs used in opioid dependence | 1.0 | 1.3 | 0.9 | 0.8 | 1.0 | 1.3 | 1.2 |
| Assessed during the year before and not including the index date, % |  |  |  |  |  |  |  |
| Antihypertensives | 70.7 | 67.5 | 63.9 | 65.3 | 76.6 | 75.9 | 73.9 |
| Cholesterol-lowering medications | 50.3 | 50.8 | 49.6 | 51.9 | 56.8 | 53.4 | 52.6 |
| Anticoagulants | 23.8 | 25.9 | 24.0 | 22.2 | 32.0 | 28.2 | 27.1 |
| Treatments for Parkinson’s disease | 7.5 | 60.4 | 59.2 | 11.1 | 6.4 | 3.0 | 8.5 |
| Anti-inflammatory drugs | 25.1 | 29.4 | 27.3 | 25.6 | 24.8 | 30.6 | 28.6 |
| Osteoporosis treatment | 9.1 | 8.4 | 8.0 | 7.9 | 7.7 | 9.1 | 8.9 |
| Bronchodilators | 12.3 | 14.0 | 12.9 | 13.4 | 13.2 | 15.0 | 14.0 |
| Oral anti-infectives | 48.8 | 51.1 | 49.0 | 47.3 | 50.3 | 49.6 | 49.4 |
| Parenteral anti-infectives | 12.4 | 14.9 | 14.2 | 13.2 | 13.4 | 14.3 | 13.7 |

AD = Alzheimer’s disease; DLB = dementia with Lewy bodies; FTD = frontotemporal dementia; PDD = Parkinson’s disease dementia; VD = vascular dementia.

1. Healthcare Utilization in Patients With Dementia-Related Psychosis Assessed in the Year Before and Not Including the Index Date

|  | AD,  n = 150,375 | PDD,  n = 49,004 | DLB,  n = 15,263 | FTD,  n = 4,382 | VD,  n = 60,132 | Unspecified dementia,  n = 269,291 | All dementia-related psychosis,  N = 484,520 |
| --- | --- | --- | --- | --- | --- | --- | --- |
| No. of hospitalizations^a^, mean (SD) | 1 (1.2) | 1 (1.2) | 1 (1.2) | 1 (1.1) | 1 (1.3) | 1 (1.3) | 1 (1.3) |
| No. of emergency department visits^b^, mean (SD) | 2 (2.0) | 2 (2.1) | 2 (2.1) | 2 (2.1) | 2 (2.2) | 2 (2.2) | 2 (2.1) |
| No. of unique days on which a clinic visit occurred^c^, mean (SD) | 8 (7.5) | 11 (8.6) | 10 (8.2) | 10 (8.5) | 8 (7.7) | 10 (8.3) | 9 (8.2) |

AD = Alzheimer’s disease; DLB = dementia with Lewy bodies; FTD = frontotemporal dementia; MedPAR = Medicare Provider Analysis and Review; PDD = Parkinson’s disease dementia; SD = standard deviation; VD = vascular dementia.

^a^ Defined as the number of unique hospital admissions evaluated in the Medicare MedPAR files.

^b^ Identified as the number of unique days with an emergency department visit from the Medicare MedPAR files where emergency room charge amount was > $0 or in the Outpatient file where the revenue center code indicated an emergency department visit.

^c^ Evaluated using outpatient and physician billing claims.

1. Incidence Rates of Events of Interest in Patients With Dementia-Related Psychosis

| Outcome | No. of patients^a^ | No. of events | Person-years | Incidence rate per 100 person-years (95% CI) ^b^ |
| --- | --- | --- | --- | --- |
| Composite falls/fractures |  |  |  |  |
| Alzheimer’s disease | 64,673 | 136,702 | 242,064.21 | 56.47 (56.17-56.77) |
| Parkinson’s disease dementia | 22,315 | 51,671 | 78,096.11 | 66.16 (65.59-66.73) |
| Dementia with Lewy bodies | 6,798 | 15,242 | 23,007.23 | 66.25 (65.20-67.30) |
| Frontotemporal dementia | 1,809 | 3,909 | 6,905.14 | 56.61 (54.84-58.38) |
| Vascular dementia | 24,425 | 51,095 | 88,705.65 | 57.60 (57.10-58.10) |
| Dementia, unspecified | 115,511 | 248,816 | 499,324.95 | 49.83 (49.63-50.03) |
| All dementia-related psychosis | 208,427 | 448,761 | 844,372.91 | 53.15 (52.99-53.30) |
| Aspiration pneumonia |  |  |  |  |
| Alzheimer’s disease | 16,807 | 22,909 | 243,244.12 | 9.42 (9.30-9.54) |
| Parkinson’s disease dementia | 7,399 | 10,951 | 78,376.57 | 13.97 (13.71-14.23) |
| Dementia with Lewy bodies | 2,423 | 3,698 | 23,056.87 | 16.04 (15.52-16.56) |
| Frontotemporal dementia | 470 | 671 | 6,935.97 | 9.67 (8.94-10.41) |
| Vascular dementia | 7,506 | 10,482 | 89,005.90 | 11.78 (11.55-12.00) |
| Dementia, unspecified | 26,864 | 35,818 | 501,836.12 | 7.14 (7.06-7.21) |
| All dementia-related psychosis | 52,327 | 71,194 | 848,465.34 | 8.39 (8.33-8.45) |
| Serious hospitalized infection |  |  |  |  |
| Alzheimer’s disease | 46,964 | 67,254 | 239,912.70 | 28.03 (27.82-28.24) |
| Parkinson’s disease dementia | 16,679 | 24,983 | 77,305.46 | 32.32 (31.92-32.72) |
| Dementia with Lewy bodies | 5,101 | 7,615 | 22,754.90 | 33.47 (32.71-34.22) |
| Frontotemporal dementia | 1,229 | 1,733 | 6,859.58 | 25.26 (24.07-26.45) |
| Vascular dementia | 19,770 | 29,236 | 87,587.91 | 33.38 (33.00-33.76) |
| Dementia, unspecified | 93,088 | 139,802 | 494,068.96 | 28.30 (28.15-28.44) |
| All dementia-related psychosis | 162,014 | 240,051 | 835,811.72 | 28.72 (28.61-28.84) |
| Parenteral anti-infective episodes |  |  |  |  |
| Alzheimer’s disease | 29,217 | 47,729 | 244,751.85 | 19.50 (19.33-19.68) |
| Parkinson’s disease dementia | 11,186 | 19,552 | 79,112.90 | 24.71 (24.37-25.06) |
| Dementia with Lewy bodies | 3,179 | 5,443 | 23,308.17 | 23.35 (22.73-23.97) |
| Frontotemporal dementia | 813 | 1,284 | 6,981.52 | 18.39 (17.39-19.40) |
| Vascular dementia | 11,518 | 19,838 | 89,711.41 | 22.11 (21.81-22.42) |
| Dementia, unspecified | 63,514 | 112,870 | 504,198.55 | 22.39 (22.26-22.52) |
| All dementia-related psychosis | 107,044 | 185,733 | 853,175.15 | 21.77 (21.67-21.87) |
| Skilled nursing facility admissions |  |  |  |  |
| Alzheimer’s disease | 68,756 | 119,062 | 230,002.96 | 51.77 (51.47-52.06) |
| Parkinson’s disease dementia | 23,307 | 43,055 | 74,144.63 | 58.07 (57.52-58.62) |
| Dementia with Lewy bodies | 7,158 | 12,907 | 21,815.06 | 59.17 (58.14-60.19) |
| Frontotemporal dementia | 1,749 | 2,961 | 6,643.09 | 44.57 (42.97-46.18) |
| Vascular dementia | 29,161 | 52,597 | 83,313.56 | 63.13 (62.59-63.67) |
| Dementia, unspecified | 124,771 | 229,327 | 479,305.54 | 47.85 (47.65-48.04) |
| All dementia-related psychosis | 225,123 | 406,928 | 806,817.30 | 50.44 (50.28-50.59) |
| Hospital admissions |  |  |  |  |
| Alzheimer’s disease | 103,304 | 230,808 | 240,101.15 | 96.13 (95.74-96.52) |
| Parkinson’s disease dementia | 34,026 | 82,694 | 77,442.26 | 106.78 (106.05-107.51) |
| Dementia with Lewy bodies | 10,550 | 24,978 | 22,777.42 | 109.66 (108.30-111.02) |
| Frontotemporal dementia | 2,903 | 6,449 | 6,843.27 | 94.24 (91.94-96.54) |
| Vascular dementia | 42,049 | 98,460 | 87,624.40 | 112.37 (111.66-113.07) |
| Dementia, unspecified | 195,471 | 487,412 | 494,916.70 | 98.48 (98.21-98.76) |
| All dementia-related psychosis | 344,695 | 830,778 | 836,917.79 | 99.27 (99.05-99.48) |
| Home healthcare episodes |  |  |  |  |
| Alzheimer’s disease | 56,021 | 111,964 | 220,124.93 | 50.86 (50.57-51.16) |
| Parkinson’s disease dementia | 21,635 | 45,818 | 68,626.48 | 66.76 (66.15-67.38) |
| Dementia with Lewy bodies | 6,476 | 13,482 | 20,269.45 | 66.51 (65.39-67.64) |
| Frontotemporal dementia | 1,683 | 3,413 | 6,272.16 | 54.42 (52.59-56.24) |
| Vascular dementia | 21,707 | 43,460 | 80,171.56 | 54.21 (53.70-54.72) |
| Dementia, unspecified | 114,532 | 229,512 | 455,493.34 | 50.39 (50.18-50.59) |
| All dementia-related psychosis | 197,938 | 398,088 | 767,332.20 | 51.88 (51.72-52.04) |
| Emergency department visits |  |  |  |  |
| Alzheimer’s disease | 122,695 | 443,305 | 244,751.85 | 181.12 (180.59-181.66) |
| Parkinson’s disease dementia | 40,339 | 162,033 | 79,112.90 | 204.81 (203.82-205.81) |
| Dementia with Lewy bodies | 12,529 | 48,616 | 23,308.17 | 208.58 (206.73-210.43) |
| Frontotemporal dementia | 3,529 | 12,734 | 6,981.52 | 182.40 (179.23-185.56) |
| Vascular dementia | 48,629 | 179,600 | 89,711.41 | 200.20 (199.27-201.12) |
| Dementia, unspecified | 228,026 | 930,745 | 504,198.55 | 184.60 (184.22-184.97) |
| All dementia-related psychosis | 404,212 | 1,587,253 | 853,175.15 | 186.04 (185.75-186.33) |
| Death |  |  |  |  |
| Alzheimer’s disease | 84,452 | 84,452 | 244,751.85 | 34.51 (34.27-34.74) |
| Parkinson’s disease dementia | 26,400 | 26,400 | 79,112.90 | 33.37 (32.97-33.77) |
| Dementia with Lewy bodies | 8,839 | 8,839 | 23,308.17 | 37.92 (37.13-38.71) |
| Frontotemporal dementia | 2,118 | 2,118 | 6,981.52 | 30.34 (29.05-31.63) |
| Vascular dementia | 32,997 | 32,997 | 89,711.41 | 36.78 (36.38-37.18) |
| Dementia, unspecified | 135,033 | 135,033 | 504,198.55 | 26.78 (26.64-26.92) |
| All dementia-related psychosis | 252,896 | 252,896 | 853,175.15 | 29.64 (29.53-29.76) |

CI = confidence interval.

^a^ Number of unique patients experiencing an event.

^b^ Confidence intervals were based on the Wald statistic.

1. Mean Cumulative Counts for Each Event of Interest in Patients With Dementia-Related Psychosis
2. Falls and Fractures


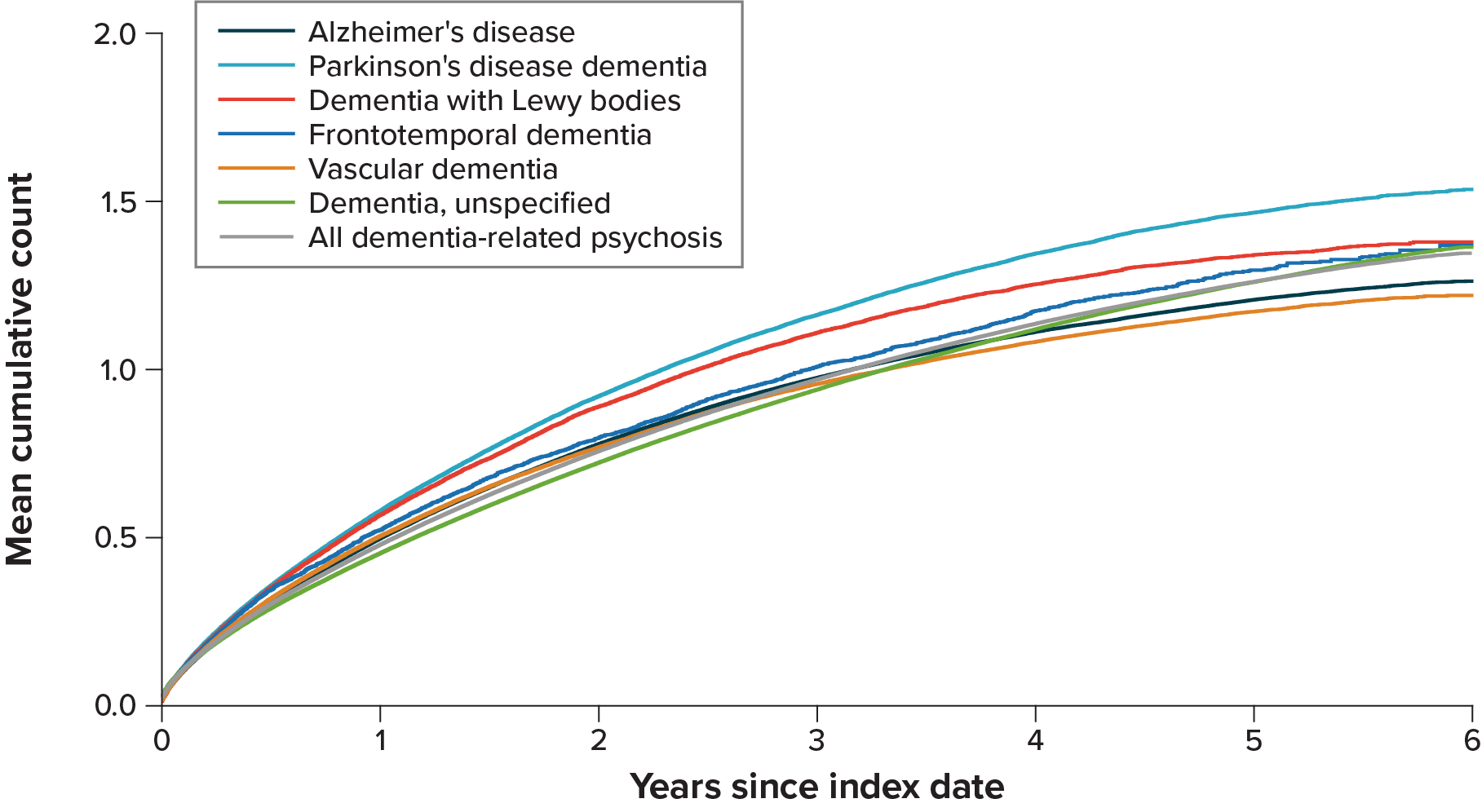


CI = confidence interval.

Alzheimer's disease: 6-year mean cumulative count = 1.3 (95% CI, 1.2-1.3)

Dementia with Lewy bodies: 6-year mean cumulative count = 1.4 (95% CI, 1.3-1.4)

Dementia, unspecified: 6-year mean cumulative count = 1.4 (95% CI, 1.4-1.4)

Frontotemporal dementia: 6-year mean cumulative count = 1.4 (95% CI, 1.3-1.5)

Parkinson's disease dementia: 6-year mean cumulative count = 1.5 (95% CI, 1.5-1.6)

Vascular dementia: 6-year mean cumulative count = 1.2 (95% CI, 1.2-1.2)

All dementia-related psychosis: 6-year mean cumulative count = 1.3 (95% CI, 1.3-1.4)

1. Aspiration Pneumonia


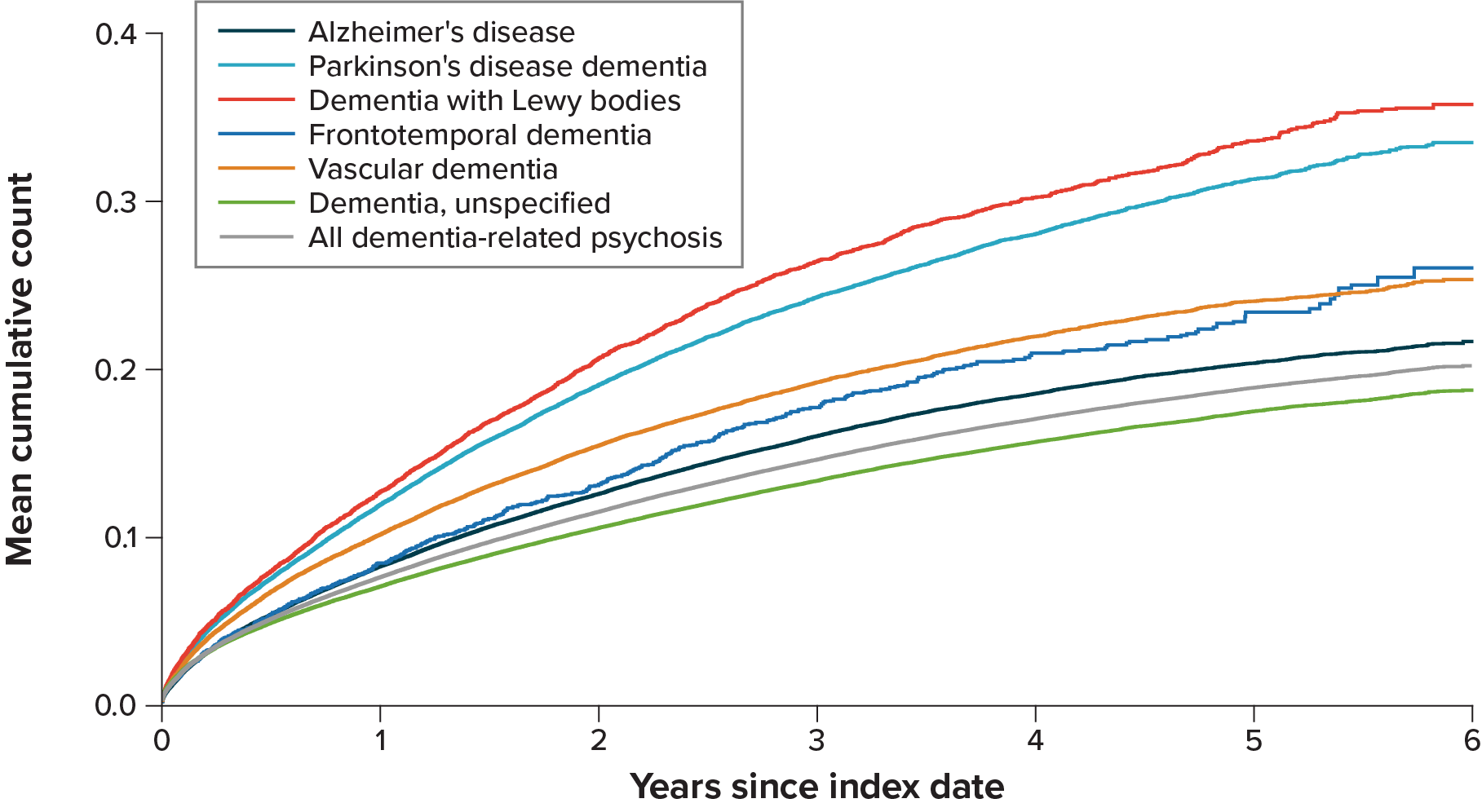


CI = confidence interval.

Alzheimer's disease: 6-year mean cumulative count = 0.2 (95% CI, 0.2-0.2)

Dementia with Lewy bodies: 6-year mean cumulative count = 0.4 (95% CI, 0.3-0.4)

Dementia, unspecified: 6-year mean cumulative count = 0.2 (95% CI, 0.2-0.2)

Frontotemporal dementia: 6-year mean cumulative count = 0.3 (95% CI, 0.2-0.3)

Parkinson's disease dementia: 6-year mean cumulative count = 0.3 (95% CI, 0.3-0.3)

Vascular dementia: 6-year mean cumulative count = 0.3 (95% CI, 0.2-0.3)

All dementia-related psychosis: 6-year mean cumulative count = 0.2 (95% CI, 0.2-0.2)

1. Serious Hospitalized Infections


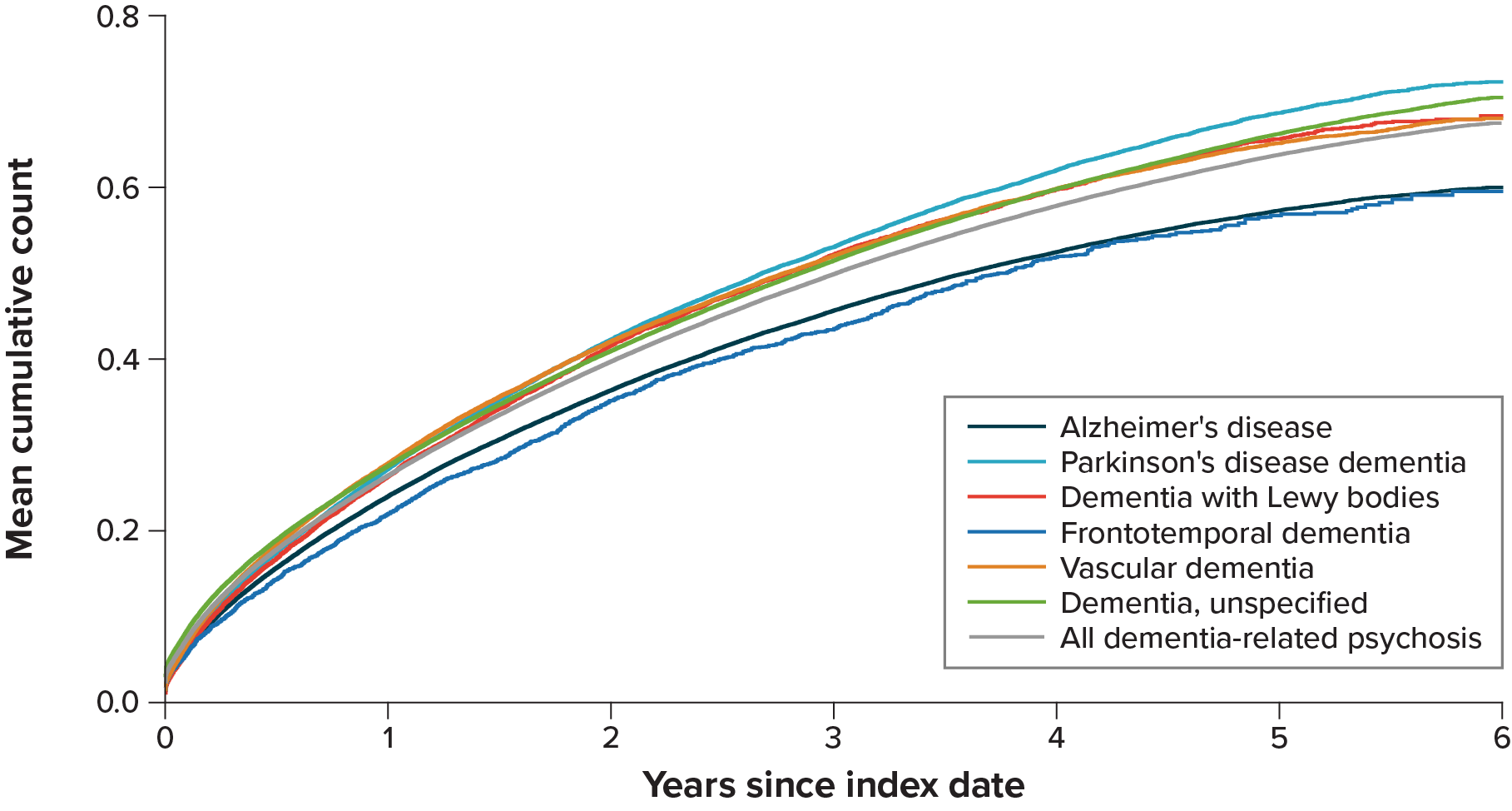


CI = confidence interval.

Alzheimer's disease: 6-year mean cumulative count = 0.6 (95% CI, 0.6-0.6)

Dementia with Lewy bodies: 6-year mean cumulative count = 0.7 (95% CI, 0.7-0.7)

Dementia, unspecified: 6-year mean cumulative count = 0.7 (95% CI, 0.7-0.7)

Frontotemporal dementia: 6-year mean cumulative count = 0.6 (95% CI, 0.6-0.6)

Parkinson's disease dementia: 6-year mean cumulative count = 0.7 (95% CI, 0.7-0.7)

Vascular dementia: 6-year mean cumulative count = 0.7 (95% CI, 0.7-0.7)

All dementia-related psychosis: 6-year mean cumulative count = 0.7 (95% CI, 0.7-0.7)

1. Parenteral Anti-infective Treatments


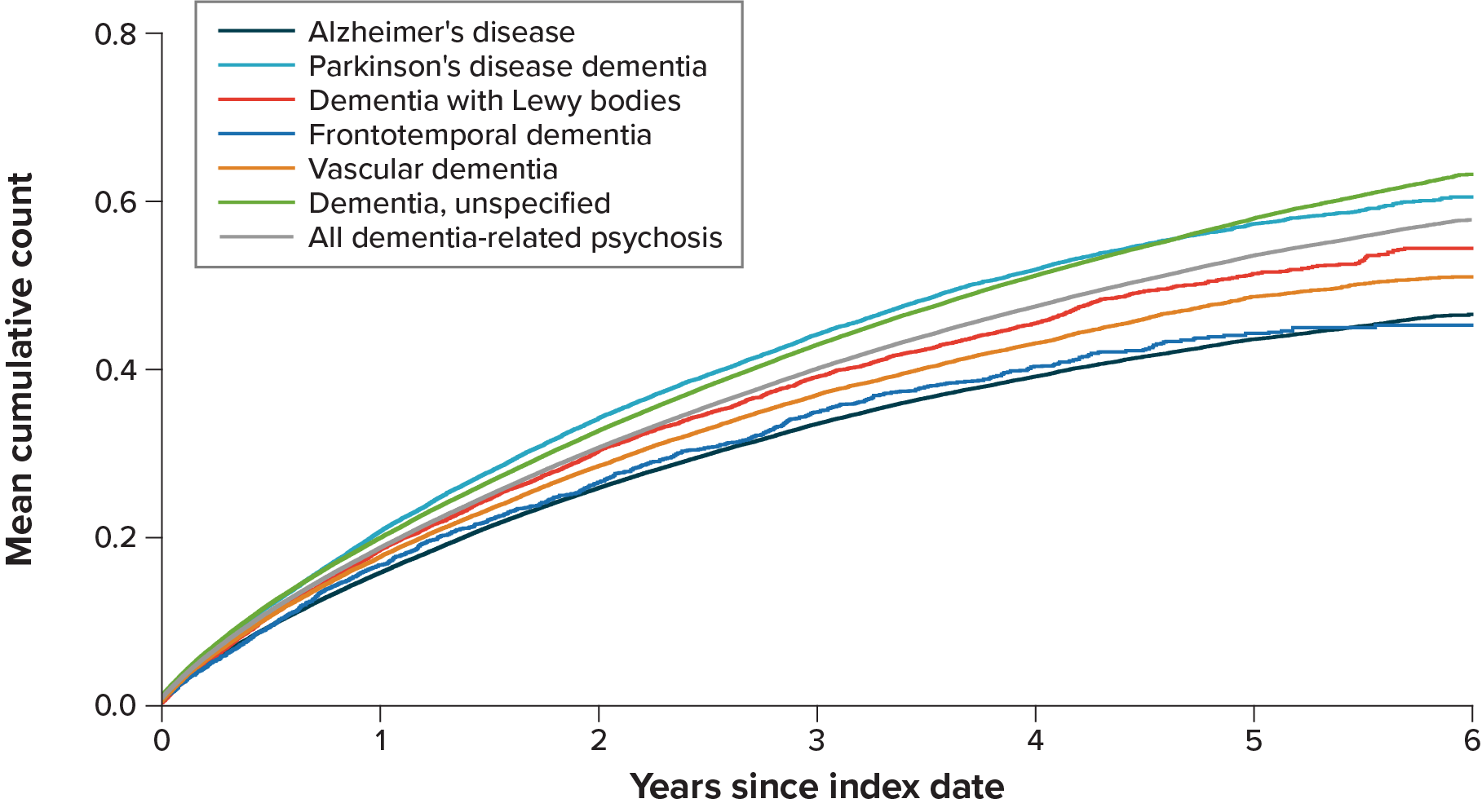


CI = confidence interval.

Alzheimer's disease: 6-year mean cumulative count = 0.5 (95% CI, 0.5-0.5)

Dementia with Lewy bodies: 6-year mean cumulative count = 0.5 (95% CI, 0.5-0.6)

Dementia, unspecified: 6-year mean cumulative count = 0.6 (95% CI, 0.6-0.6)

Frontotemporal dementia: 6-year mean cumulative count = 0.5 (95% CI, 0.4-0.5)

Parkinson's disease dementia: 6-year mean cumulative count = 0.6 (95% CI, 0.6-0.6)

Vascular dementia: 6-year mean cumulative count = 0.5 (95% CI, 0.5-0.5)

All dementia-related psychosis: 6-year mean cumulative count = 0.6 (95% CI, 0.6-0.6)

1. Skilled Nursing Facility Stays


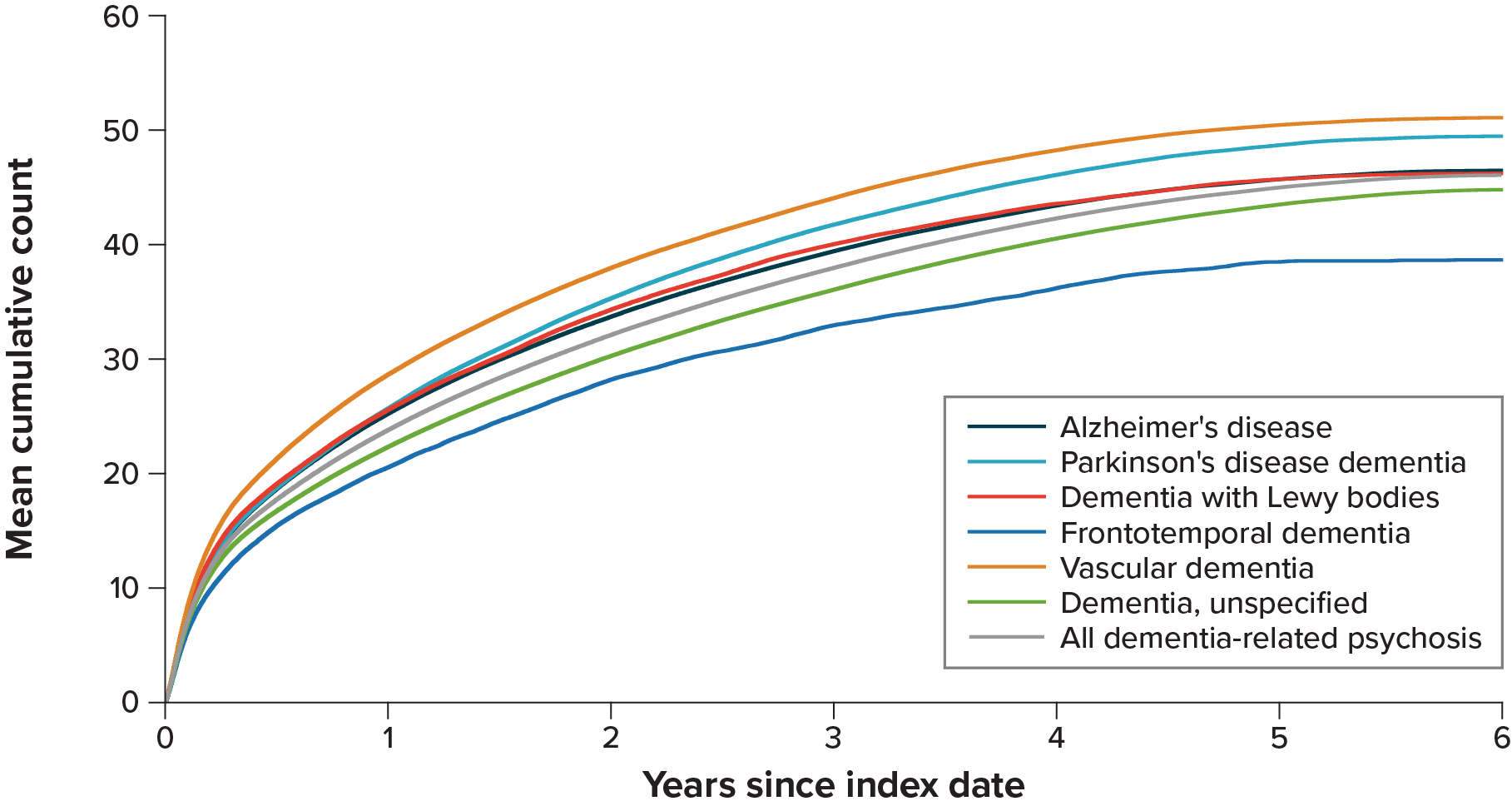


CI = confidence interval.

Alzheimer's disease: 6-year mean cumulative count = 46.5 (95% CI, 45.9-47.0)

Dementia with Lewy bodies: 6-year mean cumulative count = 46.2 (95% CI, 44.5-47.6)

Dementia, unspecified: 6-year mean cumulative count = 44.8 (95% CI, 44.3-45.1)

Frontotemporal dementia: 6-year mean cumulative count = 38.7 (95% CI, 35.8-42.4)

Parkinson's disease dementia: 6-year mean cumulative count = 49.5 (95% CI, 48.3-50.5)

Vascular dementia: 6-year mean cumulative count = 51.1 (95% CI, 50.3-52.2)

All dementia-related psychosis: 6-year mean cumulative count = 46.1 (95% CI, 45.7-46.4)

1. Days of Inpatient Hospital Stays


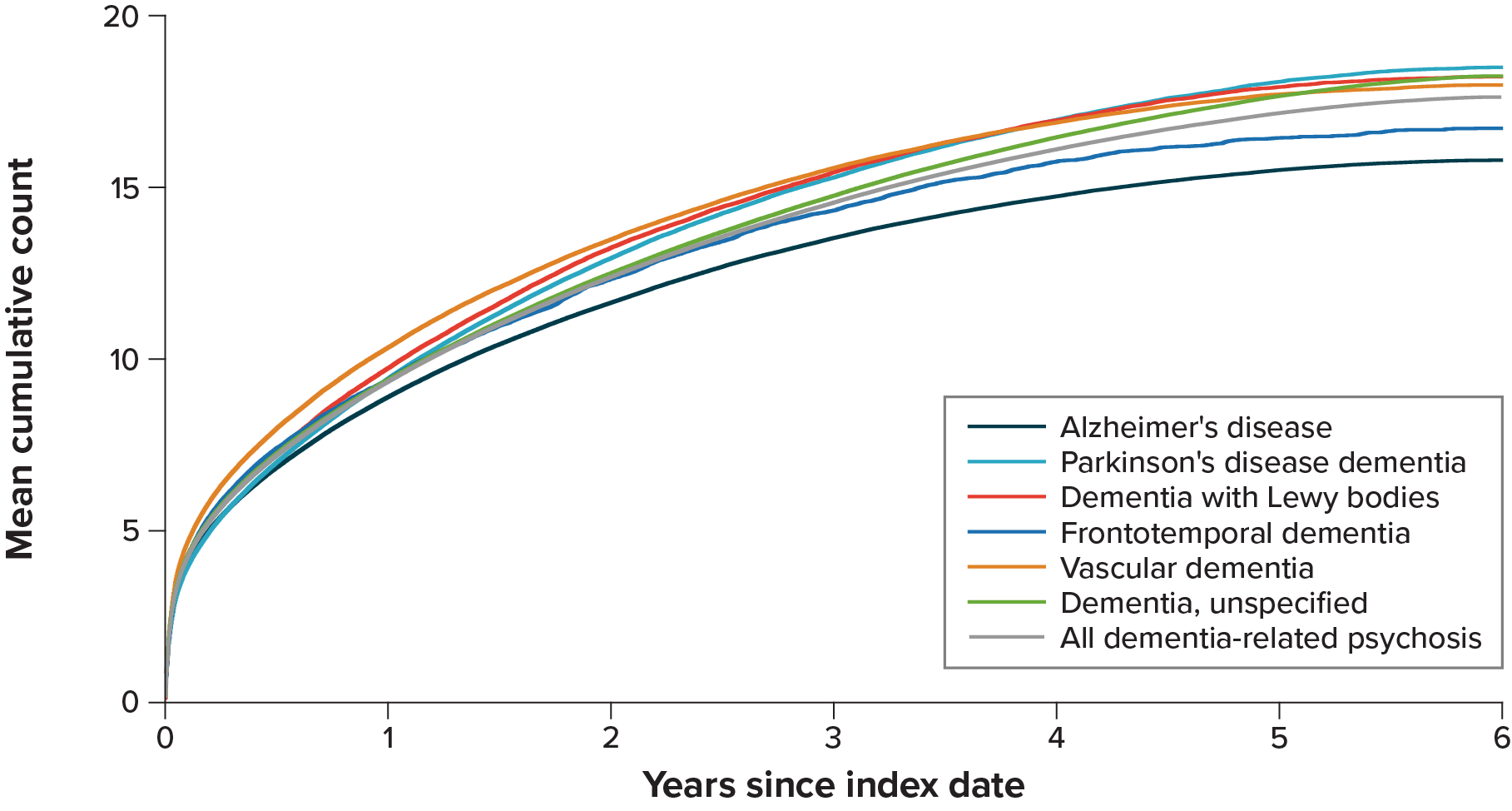


CI = confidence interval.

Alzheimer's disease: 6-year mean cumulative count = 15.8 (95% CI, 15.7-16.0)

Dementia with Lewy bodies: 6-year mean cumulative count = 18.2 (95% CI, 17.8-18.7)

Dementia, unspecified: 6-year mean cumulative count = 18.2 (95% CI, 18.1-18.4)

Frontotemporal dementia: 6-year mean cumulative count = 16.7 (95% CI, 16.0-17.5)

Parkinson's disease dementia: 6-year mean cumulative count = 18.5 (95% CI, 18.2-18.8)

Vascular dementia: 6-year mean cumulative count = 18.0 (95% CI, 17.8-18.2)

All dementia-related psychosis: 6-year mean cumulative count = 17.6 (95% CI, 17.6-17.7)

1. Home Healthcare Episodes


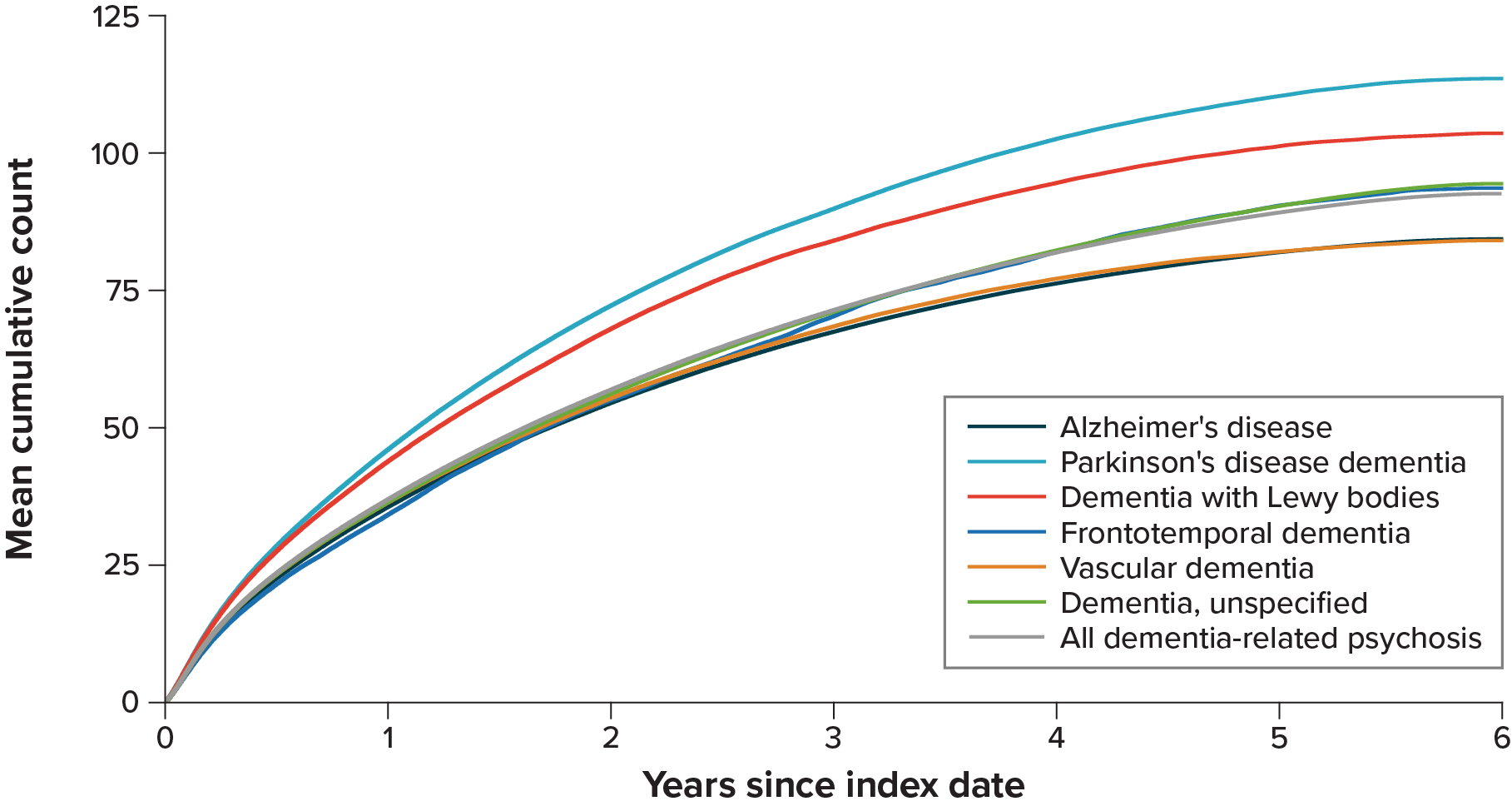


CI = confidence interval.

Alzheimer's disease: 6-year mean cumulative count = 84.4 (95% CI, 83.2-85.6)

Dementia with Lewy bodies: 6-year mean cumulative count = 103.6 (95% CI, 100.2-108.0)

Dementia, unspecified: 6-year mean cumulative count = 94.4 (95% CI, 93.5-95.4)

Frontotemporal dementia: 6-year mean cumulative count = 93.6 (95% CI, 85.6-102.5)

Parkinson's disease dementia: 6-year mean cumulative count = 113.5 (95% CI, 111.2-115.9)

Vascular dementia: 6-year mean cumulative count = 84.1 (95% CI, 82.3-86.0)

All dementia-related psychosis: 6-year mean cumulative count = 92.6 (95% CI, 91.7-93.3)

1. Emergency Department Visits


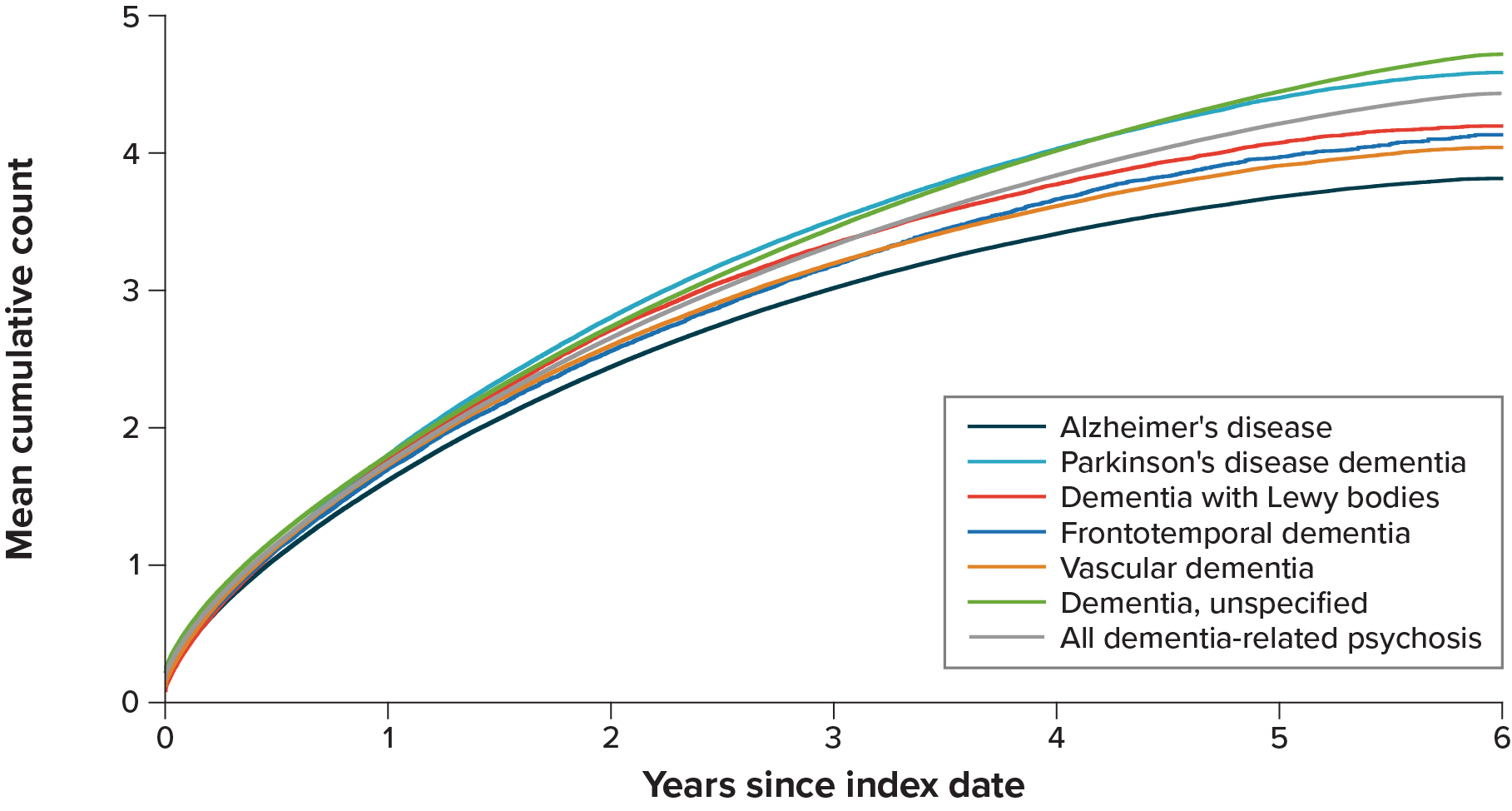


CI = confidence interval.

Alzheimer's disease: 6-year mean cumulative count = 3.8 (95% CI, 3.8-3.8)

Dementia with Lewy bodies: 6-year mean cumulative count = 4.2 (95% CI, 4.1-4.3)

Dementia, unspecified: 6-year mean cumulative count = 4.7 (95% CI, 4.7-4.7)

Frontotemporal dementia: 6-year mean cumulative count = 4.1 (95% CI, 3.9-4.4)

Parkinson's disease dementia: 6-year mean cumulative count = 4.6 (95% CI, 4.5-4.6)

Vascular dementia: 6-year mean cumulative count = 4.0 (95% CI, 4.0-4.1)

All dementia-related psychosis: 6-year mean cumulative count = 4.4 (95% CI, 4.4-4.5)

1. Characteristics of Initial Antipsychotic Treatment in Patients With Dementia-Related Psychosis

| Comedication | AD,  n = 50,164 | PDD,  n ≈ 14,970 | DLB,  n ≈ 5,280 | FTD,  n ≈ 1,610 | VD,  n ≈ 18,720 | Unspecified dementia,  n ≈ 64,130 | All dementia-related psychosis,  n = 134,022 |
| --- | --- | --- | --- | --- | --- | --- | --- |
| Time to treatment initiation with any antipsychotic after index date |  |  |  |  |  |  |  |
| Mean (SD), days | 198 (317.6) | 218 (329.7) | 181 (293.8) | 178 (315.1) | 180 (301.4) | 278 (395.7) | 238 (361.0) |
| Type of antipsychotic initially prescribed, n (%) |  |  |  |  |  |  |  |
| Clozapine | 18 (0.0) | 40 (0.3) | 16 (0.3) | <11 | <11 | <11 | 63 (0.0) |
| Quetiapine | 24,219 (49.0) | 9,389 (63.3) | 3,413 (65.4) | 834 (52.5) | 8,667 (46.9) | 29,373 (46.3) | 64,577 (48.8) |
| Olanzapine | 5,664 (11.5) | 990 (6.7) | 397 (7.6) | 173 (10.9) | 2,275 (12.3) | 6,880 (10.9) | 14,344 (10.8) |
| Risperidone | 12,784 (25.9) | 2,095 (14.1) | 746 (14.3) | 367 (23.1) | 4,773 (25.8) | 15,838 (25.0) | 32,289 (24.4) |
| Aripiprazole | 1,513 (3.1) | 366 (2.5) | 118 (2.3) | 59 (3.7) | 663 (3.6) | 2,569 (4.1) | 4,705 (3.6) |
| Brexpiprazole | 21 (0.0) | <11 | <11 | 0 (0.0) | <11 | 29 (0.0) | 56 (0.0) |
| Pimavanserin | 227 (0.5) | 893 (6.0) | 247 (4.7) | <11 | 62 (0.3) | 52 (0.1) | 980 (0.7) |
| Other atypical antipsychotic | 261 (0.5) | 54 (0.4) | 16 (0.3) | 11 (0.7) | 110 (0.6) | 285 (0.4) | 640 (0.5) |
| Haloperidol | 4,236 (8.6) | 798 (5.4) | 257 (4.9) | 122 (7.7) | 1,643 (8.9) | 5,759 (9.1) | 11,270 (8.5) |
| Other conventional antipsychotic | 1,221 (2.5) | 342 (2.3) | 74 (1.4) | 32 (2.0) | 523 (2.8) | 3,344 (5.3) | 5,098 (3.9) |
| Strength, mean (SD), mg |  |  |  |  |  |  |  |
| Clozapine | 33 (22.3) | 28 (13.3) | 27 (6.3) | NR | 36 (22.4) | 47 (38.4) | 33 (22.3) |
| Quetiapine | 34 (28.7) | 32 (23.9) | 32 (25.6) | 34 (28.3) | 35 (31.1) | 33 (25.7) | 33 (27.0) |
| Olanzapine | 4 (2.7) | 4 (2.8) | 4 (2.5) | 5 (3.4) | 4 (2.7) | 4 (2.8) | 4 (2.8) |
| Risperidone | 0 (0.4) | 0 (0.4) | 0 (0.4) | 1 (0.4) | 1 (0.4) | 0 (0.4) | 0 (0.4) |
| Aripiprazole | 4 (3.7) | 4 (3.1) | 4 (3.0) | 4 (2.6) | 5 (3.4) | 4 (3.5) | 4 (3.5) |
| Brexpiprazole | 1 (0.5) | 1 (0.6) | NR | NR | 1 (0.6) | 1 (0.4) | 1 (0.5) |
| Pimavanserin | 19 (5.1) | 18 (4.6) | 18 (4.1) | 17 (0.0) | 19 (6.0) | 19 (5.1) | 18 (4.8) |
| Other atypical antipsychotic | 27 (18.8) | 28 (24.2) | 15 (13.1) | 33 (22.2) | 27 (18.9) | 25 (17.6) | 26 (18.2) |
| Haloperidol | 1 (1.4) | 1 (1.5) | 2 (1.6) | 2 (1.5) | 1 (1.3) | 1 (1.4) | 1 (1.4) |
| Other conventional antipsychotic | 10 (11.7) | 10 (5.1) | 10 (5.3) | 13 (11.5) | 11 (11.8) | 10 (6.5) | 10 (8.2) |

AD = Alzheimer’s disease; DLB = dementia with Lewy bodies; FTD = frontotemporal dementia; NR = not reported; SD = standard deviation; PDD = Parkinson’s disease dementia; VD = vascular dementia.

Note: The n for each dementia group represents the number of patients who initiated an antipsychotic during follow-up. The n’s displayed for PDD, DLB, FTD, and VD are approximations to maintain masking of cells with <11 counts, as is required by the Centers for Medicare and Medicaid Services privacy policy.

1. Antipsychotic Treatment Status Over Follow-up in Patients With Dementia-Related Psychosis
2. Alzheimer’s Disease


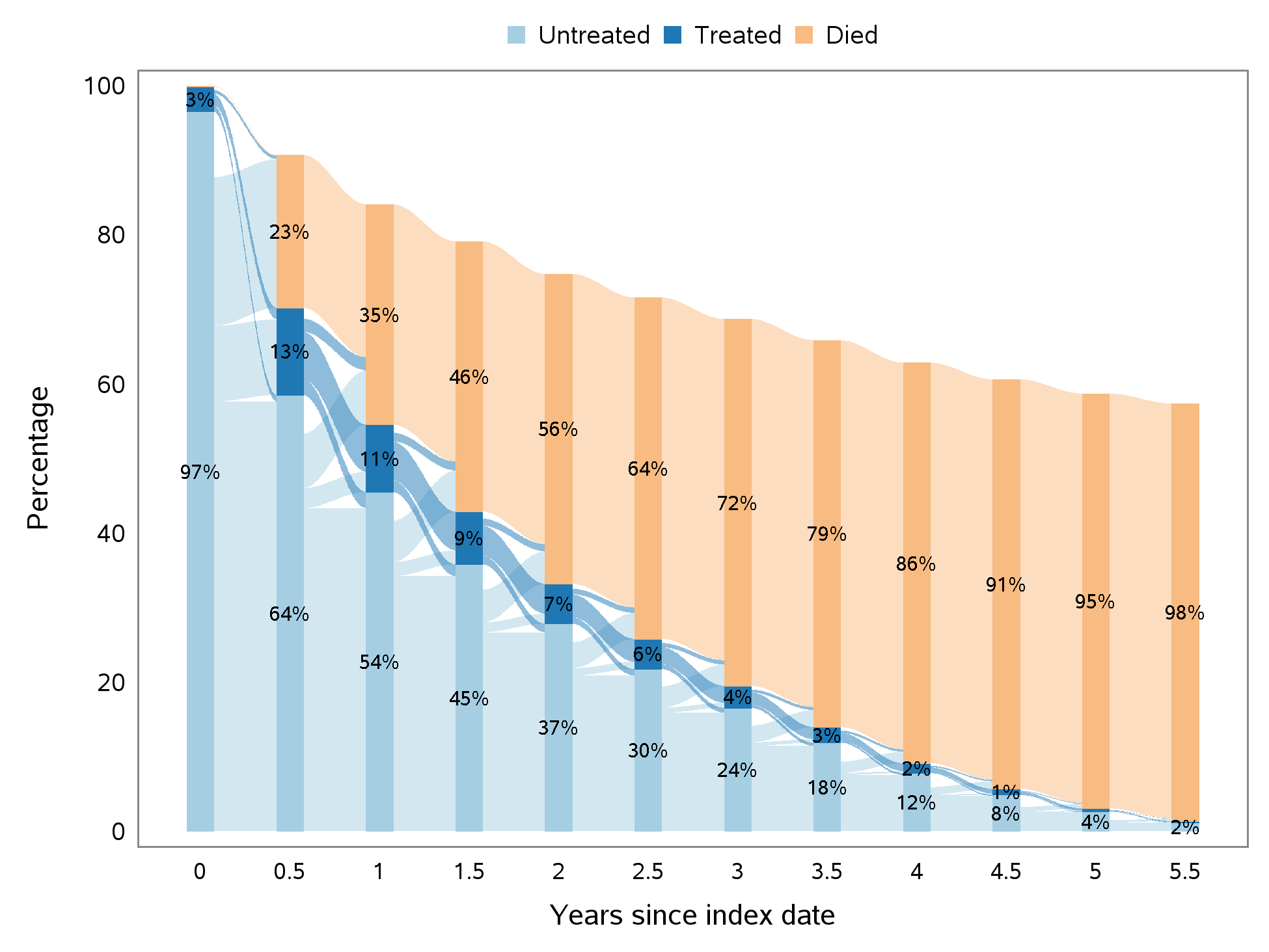


1. Parkinson’s Disease Dementia


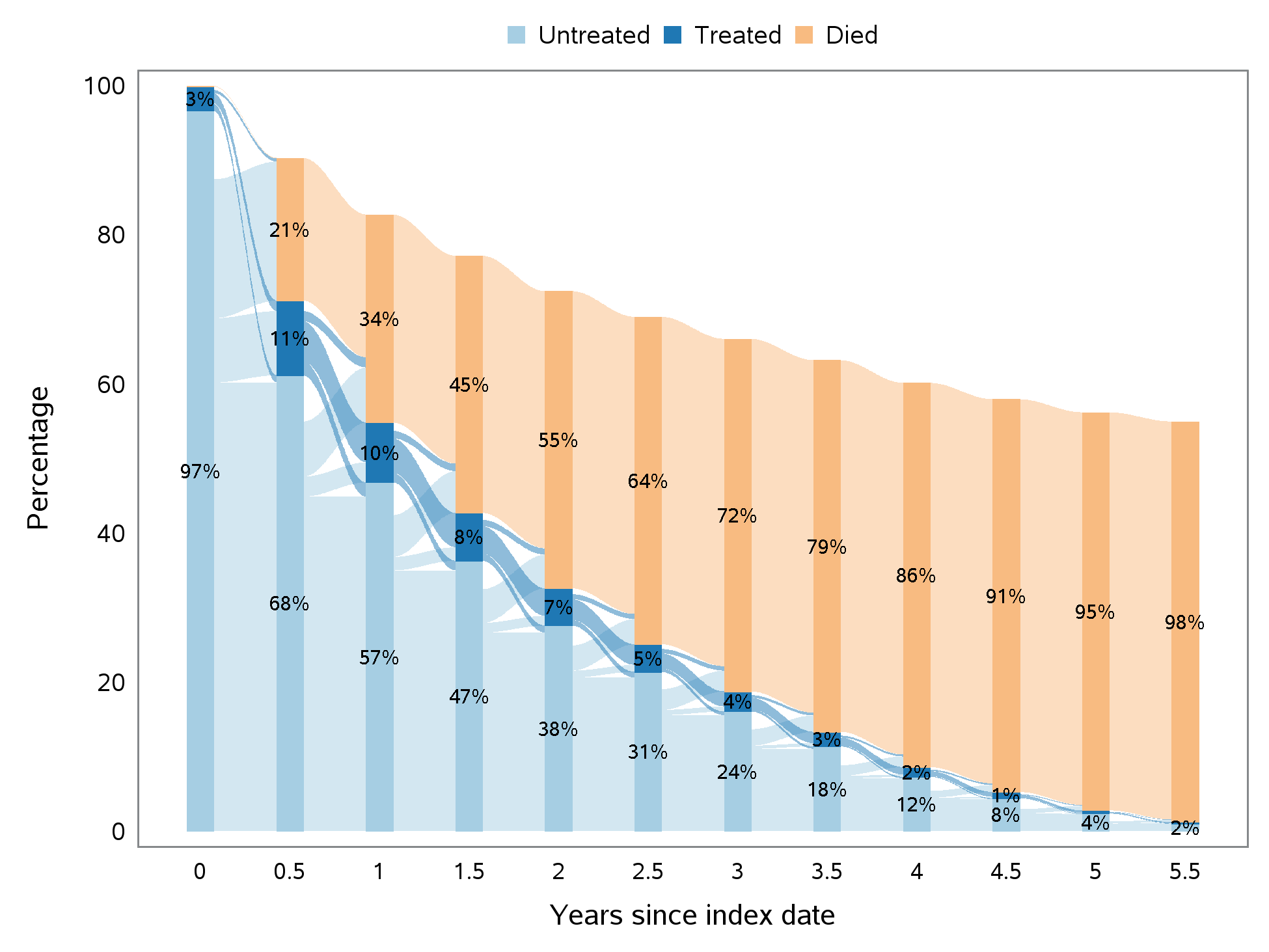


1. Dementia With Lewy Bodies


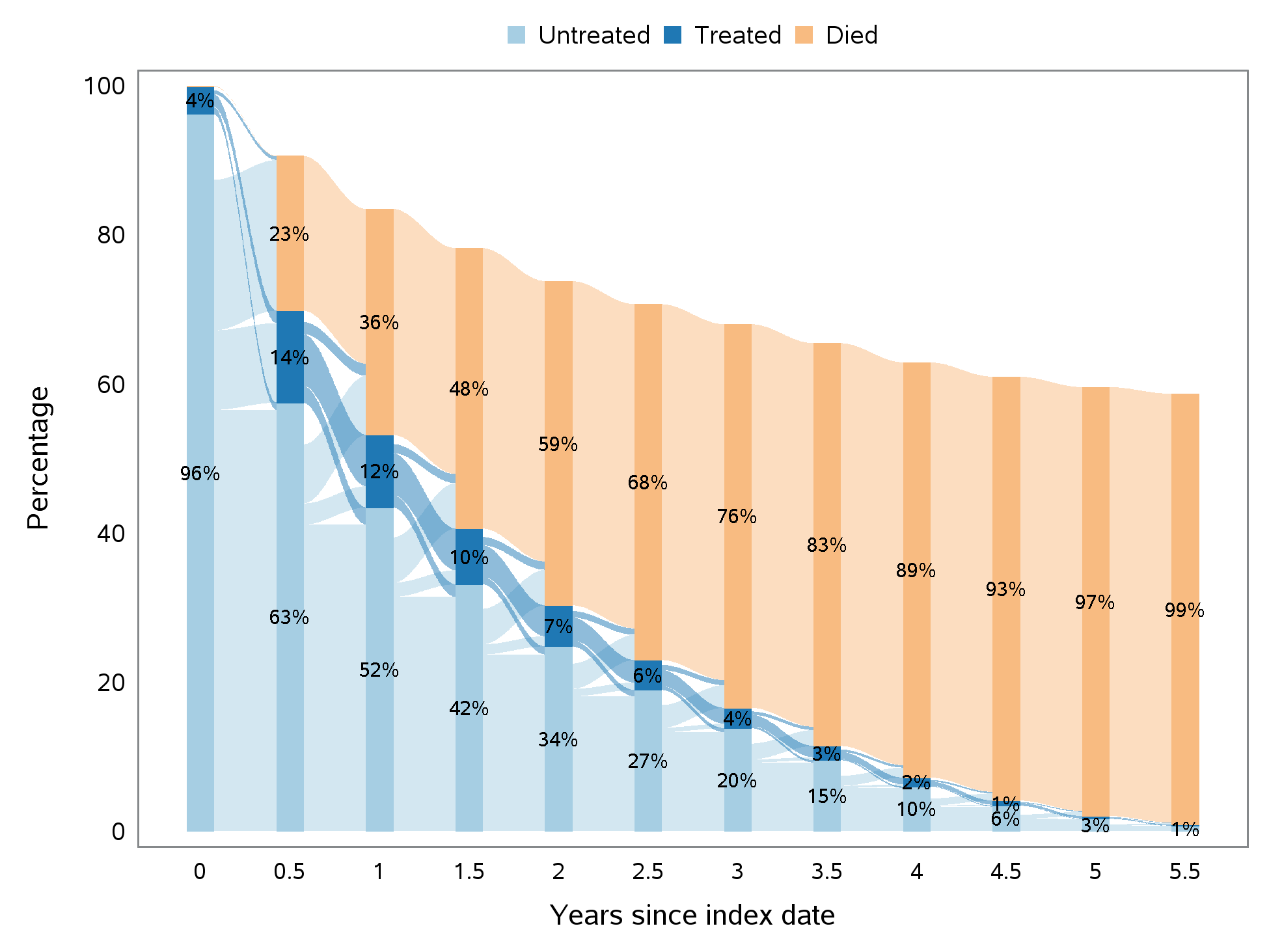


1. Frontotemporal Dementia


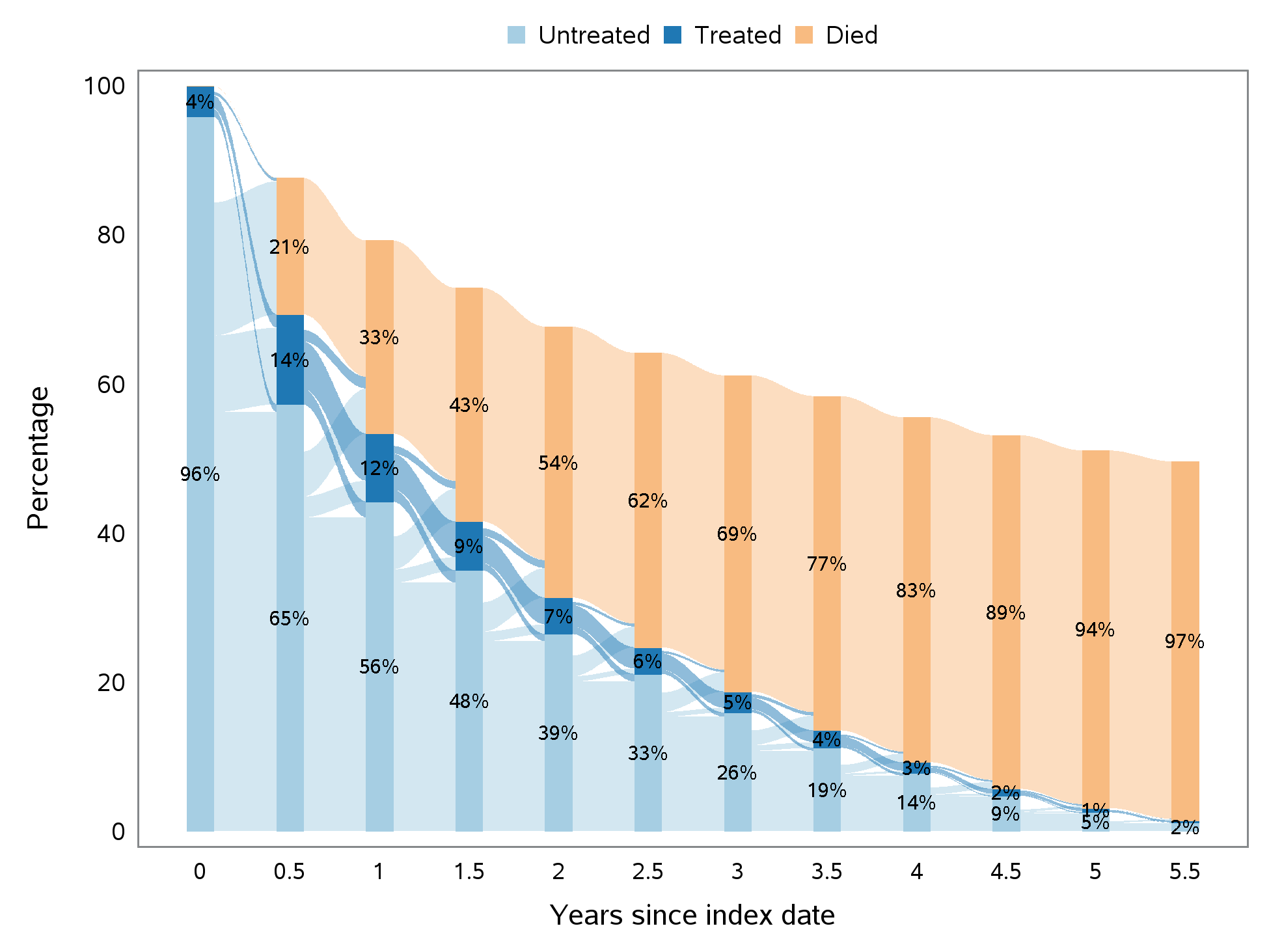


1. Vascular Dementia


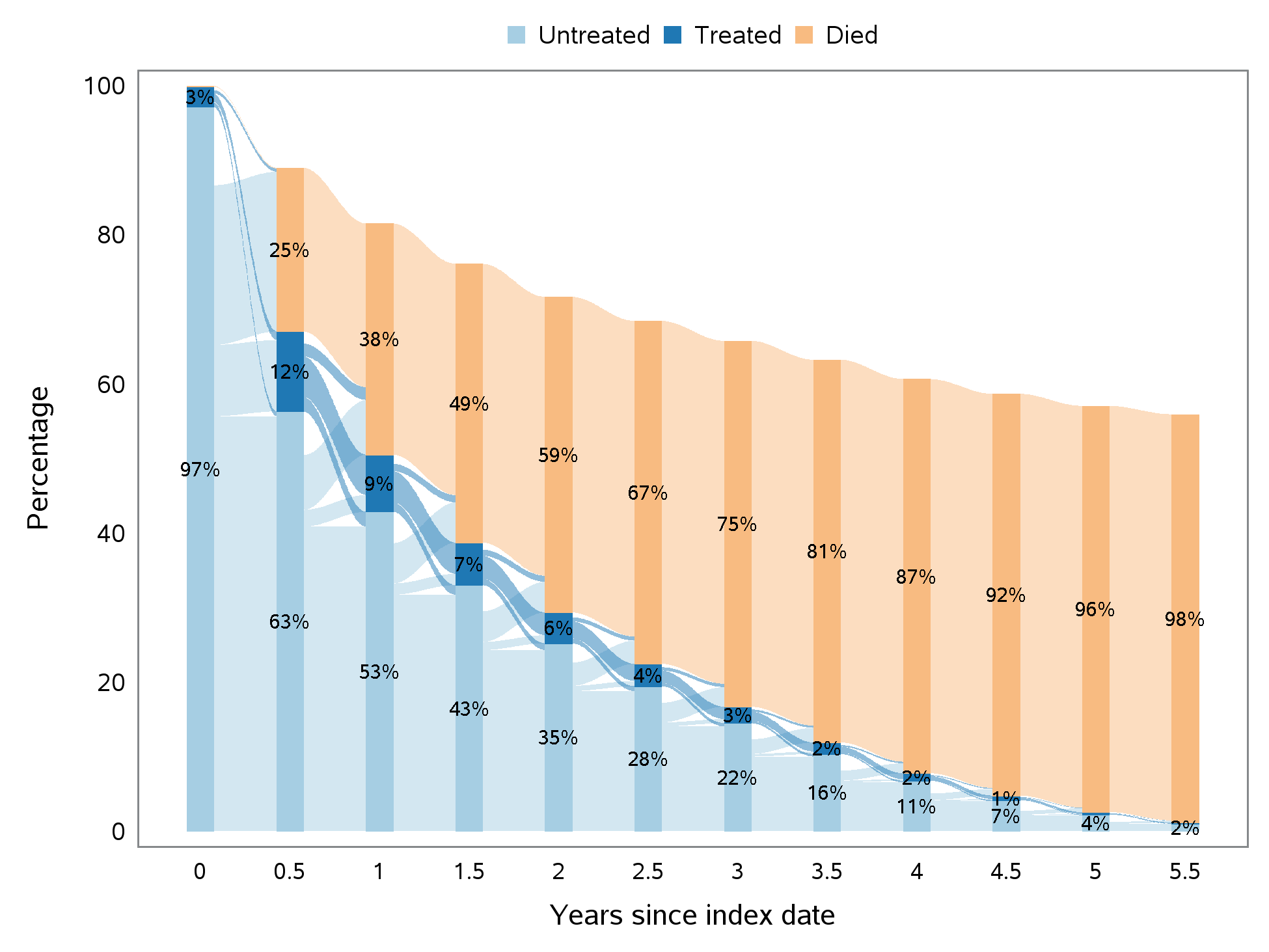


1. Dementia, Unspecified


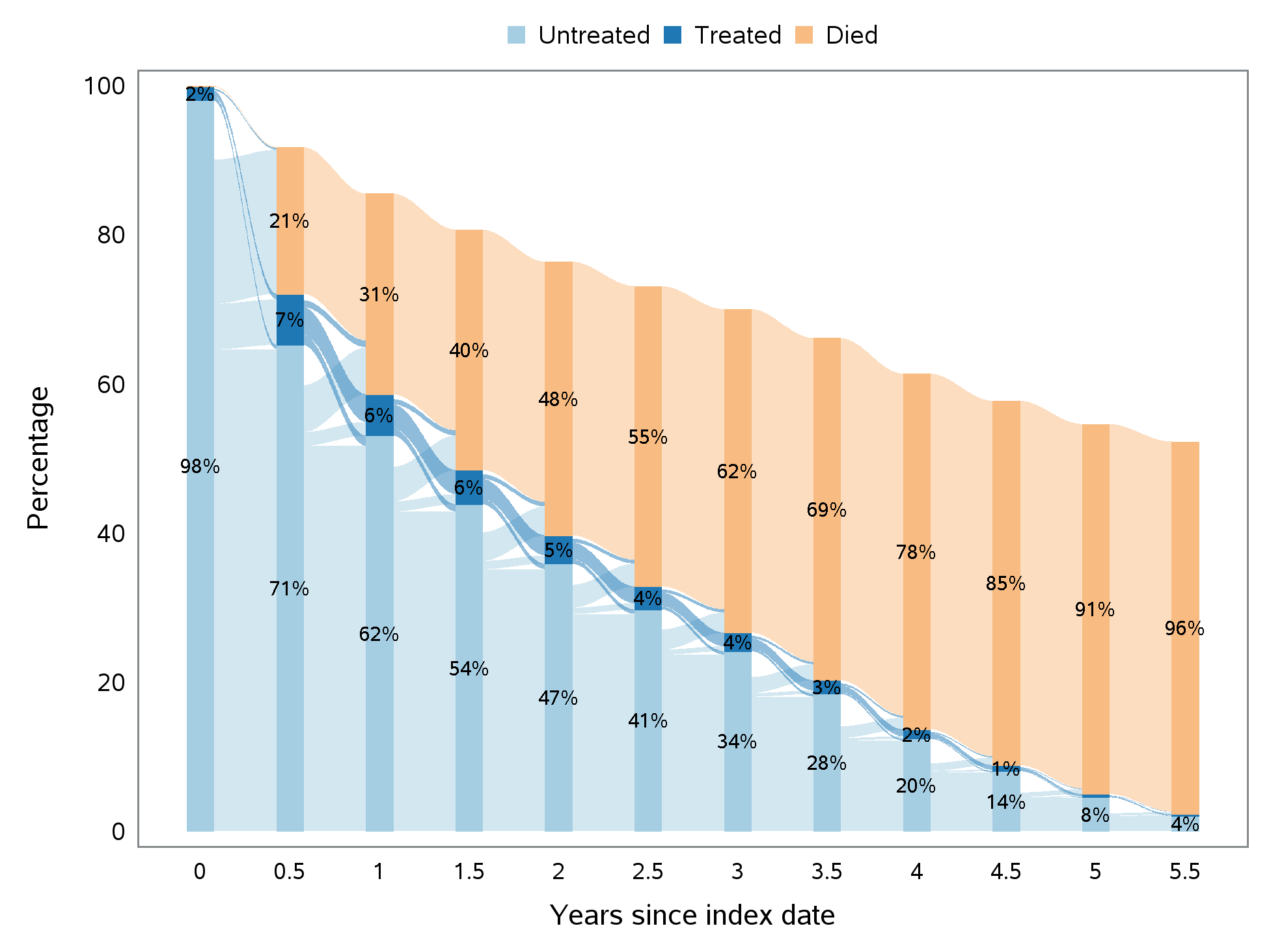


1. All Dementia-Related Psychosis


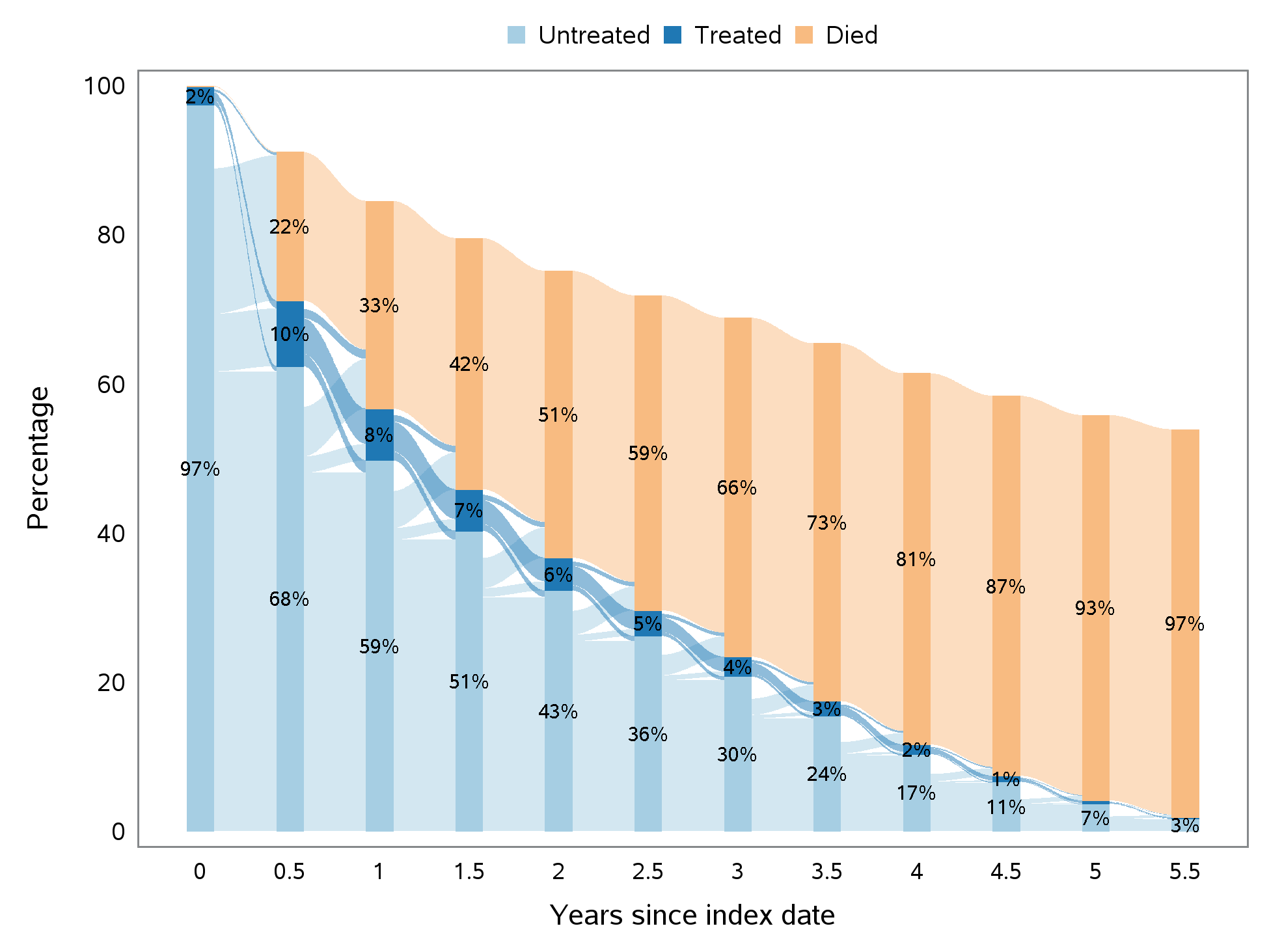


# References

1. Glasheen WP, Cordier T, Gumpina R, Haugh G, Davis J, Renda A. Charlson Comorbidity Index: ICD-9 update and ICD-10 translation. Am Health Drug Benefits. 2019 Jun-Jul;12(4):188-97.

2. Cuthbertson CC, Kucharska-Newton A, Faurot KR, Sturmer T, Jonsson Funk M, Palta P, et al. Controlling for frailty in pharmacoepidemiologic studies of older adults: validation of an existing Medicare claims–based algorithm. Epidemiology. 2018 Jul;29(4):556-61. doi:<http://dx.doi.org/10.1097/ede.0000000000000833>.

3. Faurot KR, Jonsson Funk M, Pate V, Brookhart MA, Patrick A, Hanson LC, et al. Using claims data to predict dependency in activities of daily living as a proxy for frailty. Pharmacoepidemiol Drug Saf. 2015 Jan;24(1):59-66. doi:<http://dx.doi.org/10.1002/pds.3719>.

4. Xu B, Boero IJ, Hwang L, Le QT, Moiseenko V, Sanghvi PR, et al. Aspiration pneumonia after concurrent chemoradiotherapy for head and neck cancer. Cancer. 2015 Apr 15;121(8):1303-11. doi:<http://dx.doi.org/10.1002/cncr.29207>.

5. McCarthy EP, Iezzoni LI, Davis RB, Palmer RH, Cahalane M, Hamel MB, et al. Does clinical evidence support ICD-9-CM diagnosis coding of complications? Med Care. 2000 Aug;38(8):868-76. doi:<http://dx.doi.org/10.1097/00005650-200008000-00010>.

6. Schneeweiss S, Robicsek A, Scranton R, Zuckerman D, Solomon DH. Veteran's affairs hospital discharge databases coded serious bacterial infections accurately. J Clin Epidemiol. 2007 Apr;60(4):397-409. doi:<http://dx.doi.org/10.1016/j.jclinepi.2006.07.011>.

7. Wiese AD, Griffin MR, Stein CM, Schaffner W, Greevy RA, Mitchel EF, Jr., et al. Validation of discharge diagnosis codes to identify serious infections among middle age and older adults. BMJ Open. 2018;8(6):e020857-e. doi:<http://dx.doi.org/10.1136/bmjopen-2017-020857>.

1. ICD-9-CM = *International Classification of Diseases, 9th Revision, Clinical Modification.* [↑](#footnote-ref-1)
2. ICD-10-CM = *International Classification of Diseases, 10th Revision, Clinical Modification*. [↑](#footnote-ref-2)
3. ICD-10-PCS = *International Classification of Diseases, Tenth Revision, Procedure Coding System*. [↑](#footnote-ref-3)
4. CPT = *Current Procedural Terminology*. [↑](#footnote-ref-4)
